# Time-shifted expression of acetoclastic and methylotrophic methanogenesis by a single *Methanosarcina* genomospecies predominates the methanogen dynamics in Philippine rice field soil

**DOI:** 10.1186/s40168-023-01739-z

**Published:** 2024-02-26

**Authors:** Xin Li, Qicheng Bei, Mehrdad Rabiei Nematabad, Jingjing Peng, Werner Liesack

**Affiliations:** 1 Research group “Methanotrophic Bacteria and Environmental Genomics/Transcriptomics”, Max Planck Institute for Terrestrial Microbiology, Karl-von-Frisch-Str. 10, D-35043 Marburg, Germany; 2 State Key Laboratory of Nutrient Use and Management, College of Resources and Environmental Sciences, Key Laboratory of Plant-Soil Interactions, National Academy of Agriculture Green Development, China Agricultural University, Beijing, 100193 China; 3 Present address: Institute of Agricultural and Nutritional Sciences, Martin-Luther-Universität Halle-Wittenberg, Betty-Heimann-Strasse 5, Halle (Saale), Germany; 4 Present address: Department of Soil Ecology, Helmholtz Centre for Environmental Research – UFZ, Theodor-Lieser-Strasse 4, Halle (Saale), Germany

**Keywords:** Metatranscriptomics, Metagenomics, Rice field soil, Straw, Methanogenic decomposition, Methylotrophic methanogenesis, *Methanosarcinaceae*

## Abstract

**Background:**

The final step in the anaerobic decomposition of biopolymers is methanogenesis. Rice field soils are a major anthropogenic source of methane, with straw commonly used as a fertilizer in rice farming. Here, we aimed to decipher the structural and functional responses of the methanogenic community to rice straw addition during an extended anoxic incubation (120 days) of Philippine paddy soil. The research combined process measurements, quantitative real-time PCR and RT-PCR of particular biomarkers (16S rRNA, *mcrA*), and meta-omics (environmental genomics and transcriptomics).

**Results:**

The analysis methods collectively revealed two major bacterial and methanogenic activity phases: early (days 7 to 21) and late (days 28 to 60) community responses, separated by a significant transient decline in microbial gene and transcript abundances and CH_4_ production rate. The two methanogenic activity phases corresponded to the greatest rRNA and mRNA abundances of the *Methanosarcinaceae* but differed in the methanogenic pathways expressed. While three genetically distinct *Methanosarcina* populations contributed to acetoclastic methanogenesis during the early activity phase, the late activity phase was defined by methylotrophic methanogenesis performed by a single *Methanosarcina* genomospecies. Closely related to *Methanosarcina* sp. MSH10X1, mapping of environmental transcripts onto metagenome-assembled genomes (MAGs) and population-specific reference genomes revealed this genomospecies as the key player in acetoclastic and methylotrophic methanogenesis. The anaerobic food web was driven by a complex bacterial community, with *Geobacteraceae* and *Peptococcaceae* being putative candidates for a functional interplay with *Methanosarcina*. Members of the *Methanocellaceae* were the key players in hydrogenotrophic methanogenesis, while the acetoclastic activity of *Methanotrichaceae* members was detectable only during the very late community response.

**Conclusions:**

The predominant but time-shifted expression of acetoclastic and methylotrophic methanogenesis by a single *Methanosarcina* genomospecies represents a novel finding that expands our hitherto knowledge of the methanogenic pathways being highly expressed in paddy soils.

Video Abstract

**Supplementary Information:**

The online version contains supplementary material available at 10.1186/s40168-023-01739-z.

## Background

Methane is the second most important anthropogenic greenhouse gas in terms of climate forcing, after carbon dioxide (CO_2_). It contributes to about 15% of the global anthropogenic greenhouse gases emitted yearly when assuming a greenhouse warming potential (GWP) of 25 times CO_2_ over 100 years (IPCC, 2007). Indeed, the CH_4_ emissions to the atmosphere continue to increase, with the last 3 years (2020 to 2022) having the highest annual global increase since 1983 [1].

Microbial methanogenesis by anaerobic methanogens is the largest biogenic source of atmospheric methane (CH_4_) [2]. In particular, water-logged rice paddies contribute approximately 25% to the total annual CH_4_ budget in the atmosphere [3], making them a critical source of anthropogenic CH_4_ emissions into Earth’s atmosphere. The present-day estimates of the annual emission rate from water-logged rice paddies range between 20 and 100 Tg/year [4].

Straw is one of the most abundant stocks of renewable biomass from crop production and one of the major organic carbon sources added to paddy soils as fertilizer. Rice straw, as a lignocellulosic biomass, is comprised of cellulose (32–37%), hemicellulose (29–37%), lignin (5–15%), and pectin (2–3%). The straw components serve as substrates for a complex microbial community that finally degrades the biopolymers to CO_2_ and CH_4_ [5–8]. In consequence, the anaerobic decomposition of rice straw [9] ultimately enhances CH_4_ production and emission [10]. The methanogenic degradation of rice straw involves the activity of a complex microbial food web consisting of various functional guilds, including hydrolytic, fermenting, and syntrophic bacteria, as well as methanogenic archaea. This microbial consortium fulfills a cascade of anaerobic degradation steps that involve polymer hydrolysis, fermentation, syntrophic conversion of fatty acids, homoacetogenesis, and methanogenesis [8, 11, 12].

Historically, three methanogenic pathways have been recognized to contribute to CH_4_ production (substrates in parenthesis): acetoclastic (acetate), hydrogenotrophic (H_2_ and CO_2_), and methylotrophic (methanol and other methylated compounds) methanogenesis (Fig. S1). Acetoclastic and hydrogenotrophic methanogenesis have been shown to dominate CH_4_ production at a ratio of 2:1 in rice paddies [13, 14]. The analysis of Italian rice field soil concluded that methylotrophic methanogenesis plays only a minor role in rice paddy soils [15].

Numerous studies have been conducted to understand organic matter decomposition and CH_4_ production in rice field soils [16–18]. In particular, paddy soil slurries amended with rice straw have been frequently used over the last two decades to investigate the metabolic processes involved in the anaerobic degradation of plant polymers [6, 7, 19–21]. Over recent years, this research has primarily been done on two geographically distinct paddy soils sampled in Italy and the Philippines [16, 19, 22–24]. The methanogenic communities in these two soils differ in composition and response to straw amendments [25, 26].

Studies on the Italian paddy soil already involved systems-level analyses of the methanogenic community dynamics on both rRNA (structure) and mRNA (function) levels, but only for the early stage of rice straw degradation [12]. By contrast, the research on the methanogenic community in Philippine rice field soil is limited to the analysis of total DNA using primarily PCR-based amplicon sequencing of particular biomarkers. However, this methodological approach can only address specific aspects of community potential and provides information neither on the active microbial groups nor their functional gene expression at the time of sampling [23, 24, 26, 27].

This prompted us to apply a multi-methods approach to disentangle the compositional and functional dynamics of the methanogenic community in Philippine rice field soil over an incubation period of 120 days. Anoxic paddy soil slurries amended with rice straw were used as a model system. Given the previous results on paddy soil microbial communities from various geographically distinct areas [16, 19, 28], we expected to detect a dominant expression of acetoclastic and hydrogenotrophic methanogenesis by a complex methanogen community.

Central to our research was the combination of metabolite measurements and metatranscriptomic analysis of total RNA, thereby enabling us to simultaneously unravel changes in the structure (rRNA) and function (mRNA) of the methanogenic community. In addition, we applied quantitative PCR (qPCR), reverse transcription quantitative PCR (RT-qPCR) of biomarkers (16S rRNA, *mcrA*), and targeted metagenomics to assemble draft genomes of key methanogens (Fig. S2). The observed community dynamics led us to divide the long-term incubation period into three successional phases defined as the early phase (days 3 to 21), the intermediate phase (days 21 to 28), and the late phase (days 28 to 120).

## Materials and methods

### Sample collection and experimental design

Soil was obtained from the International Rice Research Institute (IRRI) in Los Banos, Republic of the Philippines. The Philippine-IRRI is located about 66 km south of Manila (14° 09′ 45″ N, 121° 15′ 35″ E, 21 m a.s.l.) [29, 30]. The main physicochemical characteristics of the Philippine rice field soil were previously described [23, 30–32]. Soil processing followed a standard procedure, including mechanical crushing, storage of the air-dried soil at room temperature, sieving (< 2 mm) before its immediate use, and preparation of straw-amended slurries [33–35].

Briefly, soil slurries were set up in 125-ml pressure bottles by thoroughly mixing 40 g dry soil, 0.5 g rice straw (1 cm pieces), and 50 ml of deionized and autoclaved water. This amount of rice straw has been commonly used in paddy soil slurry studies [6, 12, 20, 36]. It corresponds to 37.5 t ha^−1^, which is about three times higher than under field conditions [37]. The bottles were sealed with butyl rubber stoppers and flushed with N_2_ for 15 min to establish anoxic conditions. The slurries were then incubated at 30 °C for up to 120 days.

Destructive sampling was performed after 3, 7, 11, 14, 21, 28, 35, 60, and 120 days of incubation, with three replicate slurries per time point. Slurry material was sampled from each replicate, promptly shock-frozen using liquid nitrogen, and then stored for molecular analysis at − 80 °C. Pore water samples were taken and kept at −20 ℃ until metabolite analysis. The experimental design is displayed in Fig. S2.

### Metabolite measurements

Concentrations of acetate, propionate, and butyrate in the liquid sample of the soil slurries were measured by HPLC equipped with an ion-exclusion column (Aminex HPX-87-H, BioRad, München, Germany) and coupled to a UV–Vis detector (Sykam, Fürstenfeldbruck, Germany) [38]. In addition, gas samples were taken from the same set of slurries for process measurements. A GC-8A gas chromatograph (Shimadzu, Duisburg, Germany) containing a Haysep Q column was used to measure CH_4_ and CO_2_. Data were analyzed with PeakSimple software (SRI Instruments, Bad Honnef, Germany) and calculated by linear regression [12, 36, 39].

### DNA and RNA extraction

Total DNA was extracted from soil slurries using the DNeasy® PowerLyzer® PowerSoil kit (QIAGEN, Hilden, Germany) according to the manufacturer’s instructions. Agarose gel (1%) electrophoresis and fluorometry were used to check for the integrity and quantity of each DNA extract. Fluorometric measurements were done on a Qubit 2.0 Fluorometer using the Qubit dsDNA BR Assay Kit (Thermo Fisher Scientific, MA, USA).

The RNeasy® PowerSoil Total RNA Kit (QIAGEN, Hilden, Germany) was used for the extraction of total RNA. The extraction procedure followed the manufacturer’s instructions. The RNA extracts were treated with DNase I (Ambion, Austin, USA) and purified using the RNA Clean and Concentrator kit (Zymo Research, CA, USA). The integrity of purified RNA was assessed using the Experion^TM^ RNA HighSens Analysis Kit (Bio-Rad, CA, USA), while the yield was determined with the Qubit^TM^ RNA HS Assay Kit (Thermo Fisher Scientific, MA, USA).

### Quantitative PCR (qPCR) and reverse transcription qPCR (RT-qRCR)

Primer sets and temperature profiles for quantitation of bacterial 16S rRNA and *mcrA* genes (qPCR) and their transcripts (RT-qPCR) are shown in Table S1. The *mcrA* gene is a standard biomarker to detect and quantify methanogens in environmental samples [36, 39, 40]. In RT-qPCR, randomly reverse-transcribed RNA was generated using the GoScript Reverse Transcription System (Promega, Mannheim, Germany) according to the manusfacturer’s instructions. The standard curve for quantifying bacterial 16S rRNA genes and transcripts was constructed using the genomic DNA of *Escherichia coli* (calibration range from 10 to 10^9^ copies). The standard curve for quantifying *mcrA* genes and transcripts was constructed using a *mcrA* fragment cloned into the pGEM®-T Easy plasmid (Promega, WI, USA) (calibration range from 10 to 10^8^ copies) [39]. The *mcrA* fragment used for cloning was obtained from the genomic DNA of *Methanosarcina barkeri* [41]. The qPCR reactions were carried out on an iCycler Real-Time PCR Detection System (CFX Connect^TM^, Bio-Rad). The PCR efficiency was at least 85% (*R*^2^ > 0.98). The presence of unspecific products was checked by melt curve analysis.

### Illumina library preparation and sequencing

Libraries for sequencing were prepared for both metatranscriptomic (cDNA) and metagenomic (total DNA) analysis.

#### Metatranscriptomics

Twenty-seven RNA samples (three replicate slurries × 9 time points) were subjected to cDNA library preparation using the NEBNext® Ultra II Directional RNA Library Prep Kit for Illumina® (New England Biolabs, USA). The cDNA library synthesis procedure strictly followed the manufacturer’s instructions. cDNA integrity and yield were checked on a Bio-Rad analyzer using the Experion^TM^ DNA 12K Analysis Kit (Bio-Rad). The 27 cDNA libraries were sequenced (RNA-Seq) on the NovaSeq 6000 platform in paired-end mode (2×150 bp) by Novogene Genomics Service (Novogene Co., Ltd., UK).

#### Metagenomics

DNA extracts from the triplicate slurries of a given time point were mixed in equal amounts before metagenomic library construction. A total of three metagenomic libraries (one each for three incubation periods: 21, 28, and 35 days) were constructed using the TruSeq DNA Library Prep Kit according to the manufacturer’s instructions (Illumina). The metagenomic libraries were sequenced on an Illumina HiSeq-2500 platform in paired-end mode (2×250 bp) at the Max Planck Genome Centre Cologne, Germany.

### Computational analysis of metatranscriptomic datasets

#### Pre-processing

The quality filtration of raw reads was performed using Trimmomatic [42]. Default settings were applied with the exception that the minimum sequence length was set at 100 bp. Quality control of the filtered reads was visualized using FastQC. Analysis of 16S rRNA and putative mRNA reads was carried out using a bioinformatic pipeline reported previously [12]. Briefly, reads mapping to rRNA and non-coding RNA were filtered by SortMeRNA 2.0 against SILVA (release 128) [43] and RFAM reference databases [44], respectively. The remaining reads were considered putative mRNA. The sequencing statistics are shown in Table S2.

#### Analysis of 16S rRNA

Upon extraction from the metatranscriptome, the rRNA-derived reads were assembled to near full-length 16S rRNA sequences over 40 iterations using EMIRGE with the SILVA 132 SSU rRNA database [45]. This assembly approach was done separately for the 27 rRNA sequence datasets. The assemblies were grouped into population-specific 16S rRNA sequence types using an identity cutoff of 97% [46, 47]. Taxonomic assignment was performed using BLASTN implemented in DIAMOND (v0.9.25), applying an *e*-value cutoff of 1e−5 for the database searches against the SILVA 132 SSU rRNA database. The output file by DIAMOND was further processed in MEGAN6 Ultimate Edition v6.20 (Computomics, Tübingen, Germany) for a more detailed taxonomic classification [48]. The abundance of each assembled 16S rRNA sequence type was estimated using BBMap v38.62 [49]. All nearly full-length 16S rRNA sequences (> 1200 bp), which were affiliated with the family *Methanosarcinaceae*, were extracted for phylogenetic comparison. The sequence alignment was done using MUSCLE [50] and manually refined. A neighbor-joining tree was constructed in MEGAX using 500 bootstrap replications [51]. Reference sequences were downloaded from the Genomic Taxonomic Database (GTDB) (https://gtdb.ecogenomic.org/). The neighbor-joining tree was visualized by iTOL (https://itol.embl.de/) [52]. The sequencing statistics of near-full-length 16S rRNA sequences are shown in Table S3.

#### Analysis of mRNA

Trinity (v2.2.0) was used for de novo metatranscriptome assembly [53]. Total mRNA reads from all 27 cDNA libraries were pooled into one transcriptomic dataset for subsequent contig assembly using Trinity scripts [54]. A total of 472,280 quality-checked contigs were obtained. The mRNA reads of each cDNA library were individually mapped back onto the contigs using Bowtie [55]. An FPKM matrix of mRNA reads mapped onto a particular contig was produced for all relevant contigs using RSEM [56] within Trinity (v2.2.0). Taxonomic assignment and functional annotation of the mRNA contigs were done using BLASTX implemented in DIAMOND (v0.9.25) [57], applying an *e* value cutoff of 1e−5 for database searches against NCBI’s non-redundant (nr) protein database. mRNA contigs encoding putative laccases and multicopper oxidases were identified by searches against LccED (Laccase and Multicopper Oxidase Engineering Database; [58]). To investigate the gene expression of particular metabolic pathways, related mRNA contigs were extracted using MEGAN6 Ultimate Edition [12, 48]. The mRNA sequencing statistics are shown in Table S4.

The identification of mRNA contigs encoding carbohydrate-active enzymes (CAZymes) was achieved by searches against the dbCAN database using BLASTX implemented in DIAMOND (v0.9.25) and applying the default *e* value cutoff of 1e−3. Functional CAZyme modules involved in the degradation of cellulose, xylan, chitin, and other hemicelluloses were defined by grouping related enzymatic functions based on their enzyme commission numbers. A mapping file for functional annotation was created using all available entries in dbCAN. The mapping file was stored as an indexed SQLite database and queried using a custom Python script. The resulting annotations are based on matching dbCAN top hits for mRNA contigs queried against the mapping file and defined CAZyme modules. The functional annotation of CAZyme-affiliated mRNA contigs to CAZyme functional modules was achieved using custom Python scripts described by Peng et al. (http://github.com/wegnerce/peng_et_al_2018.) [12]. Their taxonomic assignment was done using BLASTX implemented in DIAMOND (v0.9.25) against the NCBI nr protein database.

### Computational analysis of metagenomic datasets

#### Assembly and binning

Raw reads were quality-filtered using Trimmomatic v0.38 [42]. Upon quality filtration, the three metagenomes recovered from slurries after an incubation period of 21, 28, and 35 days were combined for the assembly of contigs. Metagenomic assembly and binning were done using the MetaWRAP pipeline [59]. The quality-filtered reads were first assembled to larger contigs using metaSPAdes v3.13.0 [60]. Metagenomic binning was performed with MetaBAT v2.12.1 [61] and MaxBin v2.2.5 [62], using contigs longer than 1000 bp. Completeness and contamination of metagenome-assembled genomes (MAGs) were assessed by CheckM v1.0.17 [63]. The sequencing statistics are shown in Table S5.

#### Genome annotation

Open reading frames (ORFs) in each MAG were identified using Prokka v1.14.5 [64]. The ORFs were then queried against the nr protein database using DIAMOND (v0.9.25) with an *e* value of 1e−5 [57]. Functional annotation was performed based on the Kyoto Encyclopedia of Genes and Genomes (KEGG) database using the lowest common ancestor (LCA) algorithm in MEGAN6 Ultimate Edition v6.20 [48]. FastANI was used to calculate genome average nucleotide identity (ANI) values [65]. Whole-genome sequences were visualized and compared using CGView software [66].

#### Transcript mapping onto Methanosarcina MAGs

To determine how strongly particular methanogenic pathways and key pathway genes were expressed by different *Methanosarcina* populations, forward and reverse reads of the mRNA sequences were pooled and mapped onto the MAGs using BBMap v38.62 pipeline (minimum sequence identity set to 0.97) [46, 47, 49]. Competitive mapping of the mRNA reads was done using BBSplit in BBMap v38.62 [49]. Each metatranscriptome replicate of a given sampling time point (days 3, 7, 11, 14, 21, 28, 35, 60, and 120) was mapped separately onto a mix of *Methanosarcina* genomes including *M. fluorescens* (Group I), strain MSH10X1 (Group II), *M. barkeri* (Group III), and *M. horonobensis* (Group IV), and it was determined to which genome the metatranscriptomes of a given incubation time point match best. The relative mapping efficiencies were calculated based on the normalized number of mRNA reads that were competitively mapped onto each *Methanosarcina* reference genome.

#### Phylogenetic analysis

CheckM (v1.0.17) was used to predict the 16S rRNA genes in the MAGs and for their taxonomic assignment [63]. Subsequently, a genus-level 16S rRNA gene tree for *Methanosarcina* spp. was constructed in MEGAX using the neighbor-joining algorithm and 500 bootstrap replications. Reference sequences were extracted from *Methanosarcina* genomes downloaded from GTDB.

### Statistical analysis

All means ± standard errors (SE) are based on the analytical results of three independent replicate slurries (metabolite measurements, qPCR, RT-qPCR, and metatranscriptomic community dynamics on rRNA and mRNA levels). Significant differences in the qPCR and RT-qPCR measurements across the nine incubation time points were determined using one-way ANOVA. The resulting *P* values were corrected for multiple tests using the Benjamini-Hochberg false discovery rate method (*P*_FDR_ < 0.05) [67]. In metatranscriptomic analysis, the relative abundance values calculated for taxon-specific 16S rRNA and mRNA contigs (domain and family level), and for mRNA contigs assigned to particular KEGG categories (functional gene expression analysis), were normalized to transcripts per kilobase million (TPM). This was done to normalize for varying sequencing depths. Tukey’s honest squared difference test (Tukey HSD) in STAMP was used to determine whether the taxon-specific rRNA/mRNA abundances and the expression of particular functional categories significantly differed across the 120-day incubation period (significant with *p* < 0.05) [68]. The DESeq2 package v1.24.0 in R (v3.6.1) was used to further test for significant differences in taxon-specific rRNA abundance and the mRNA abundance of key pathway genes between two sampling time points [69]. In particular, it was tested whether the taxon-specific rRNA abundance on a particular sampling time point significantly differed from the initial abundance at the first sampling (day 3) or vice versa whether the taxon-specific rRNA abundance at day 3 significantly differed from those of all other sampling time points. The same analysis approach was applied to the mRNA abundance of key pathway genes. All *P* values generated with STAMP or DESeq2 were corrected for multiple testing using the Benjamini-Hochberg method to control the false discovery rate (FDR) with *P*_*FDR*_ < 0.05.

## Results

### Metabolite turnover

**Fig. 1:**
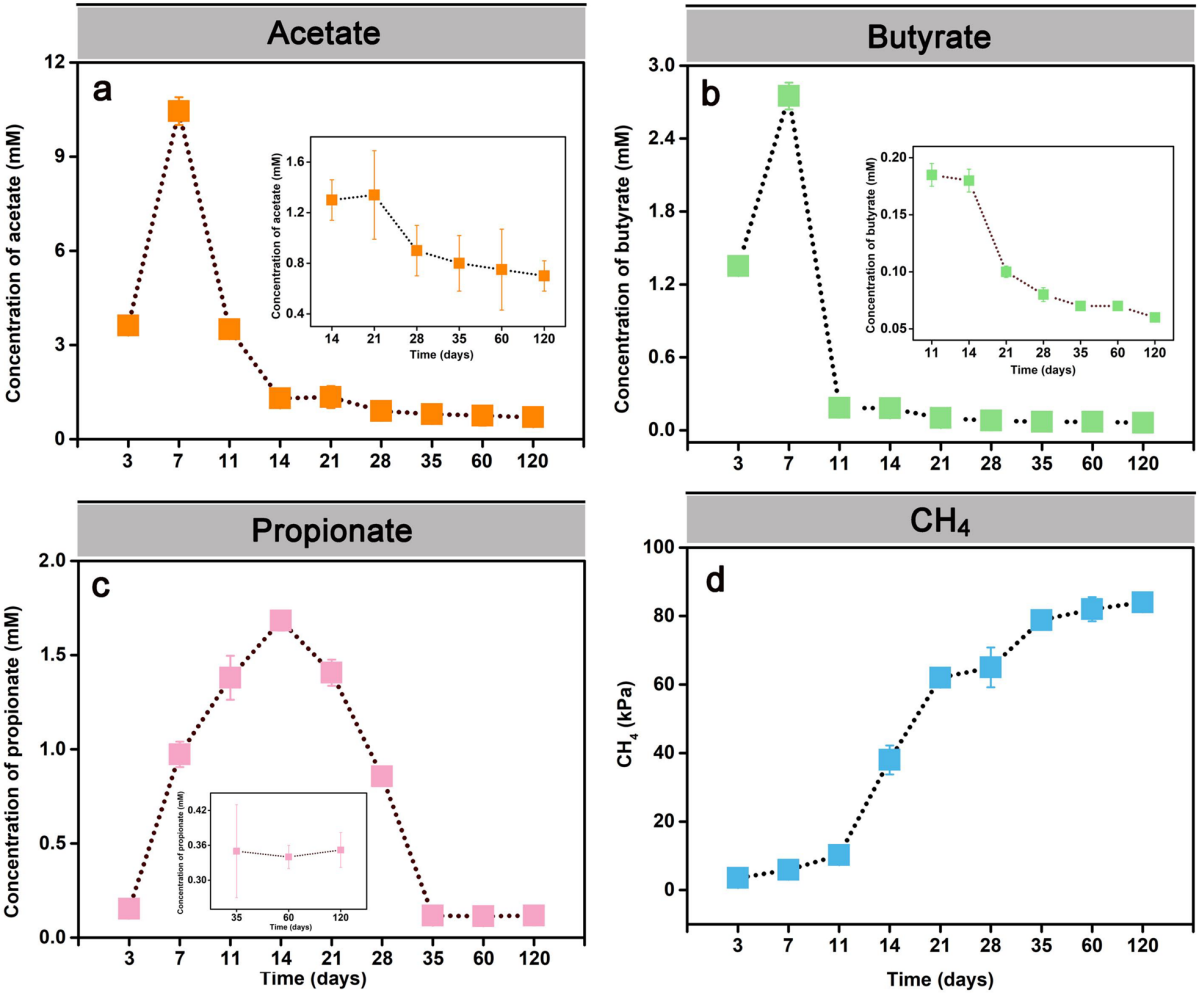
Measurement of intermediates and methane in the paddy soil slurries over the 120-day incubation period at 30 °C. Intermediate turnover of acetate **a**, butyrate **b**, and propionate **c**, and methane production **d**. The inserts show the near-steady-state concentrations of acetate for the incubation period 14 to 120 days **a**, butyrate for the incubation period 11 to 120 days **b**, and propionate for the incubation period 35 to 120 days (**c**) in high-resolution. Data are means ± SE (*n* = 3)

Metabolite measurements in the straw-amended slurries were made over the 120-day incubation period. Acetate transiently accumulated and reached its peak concentration (10.57 mM) at day 7. Thereafter, the acetate concentration decreased within 7 days to 1.42 mM on day 14 and then showed a steady state between production and consumption, ranging from 0.83 to 1.30 mM until day 120 (Fig. 1a). Concurrently, the butyrate concentration peaked on day 7 (2.88 mM) and then decreased to low but detectable steady-state levels (0.08 to 0.18 mM) (Fig. 1b). Propionate accumulated from day 3 (0.57 mM) onwards, with a peak concentration on day 14 (1.68 mM). Subsequently, its concentration decreased towards day 35 (0.65 mM), and then, propionate exhibited a steady state at low but clearly detectable levels (0.35 mM) (Fig. 1c). First CH_4_ production was detectable around day 3 (2.5 kPa), while its headspace concentration increased significantly towards day 21 (62.15 kPa). Then, the CH_4_ production rate was decreased between days 21 and 28 (Fig. 1d), but increased again from days 28 to 35. The greatest net CH_4_ production rate was observed between days 11 and 21, concomitantly to the decline of acetate and butyrate to low steady-state concentrations, and in part to the net consumption of propionate (Fig. 1d).

### qPCR and RT-qPCR

**Fig. 2:**
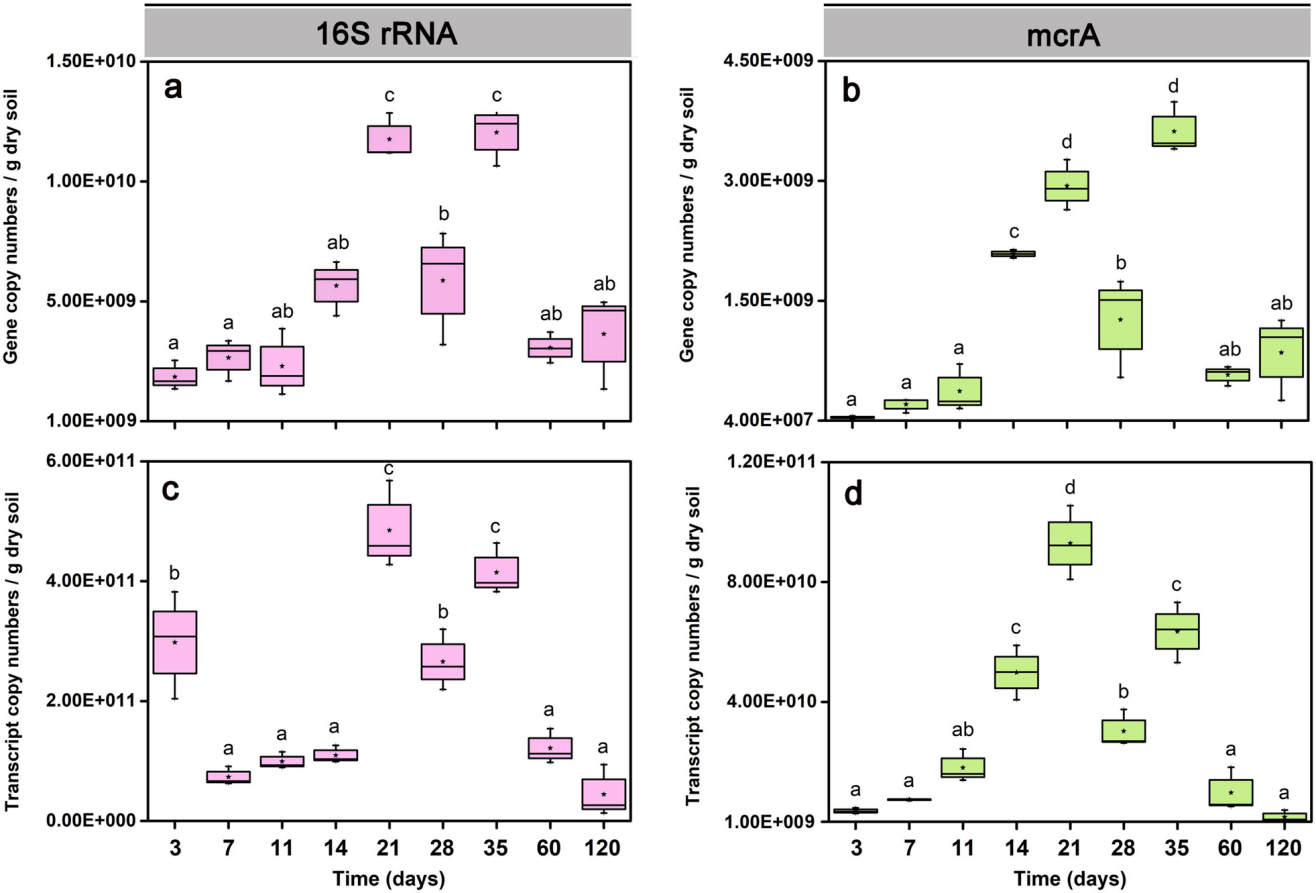
Copy number quantification of bacterial 16S rRNA and methanogenic *mcrA* genes (qPCR) and their transcripts (RT-qPCR) in Philippine paddy soil slurries over the 120-day incubation period. Copy numbers of bacterial 16S rRNA genes (**a**) and transcripts (**c**) per gram of dry paddy soil. Copy numbers of *mcrA* genes (**b**) and transcripts (**d**) per gram of dry paddy soil. Data are means ± SE (*n* = 3). Differences between two incubation time points were determined using one-way ANOVA (*P*_*FDR*_ < 0.05). The exact copy numbers per gram of dry paddy soil are shown in Tables S6, S7 (genes) and S8, S9 (transcripts)

Genes and transcripts of bacterial 16S rRNA and methanogenic *mcrA* were quantified over the 120-day incubation period (Fig. 2, Tables S6, S7, S8, S9). Changes in the copy numbers of bacterial 16S rRNA genes and transcripts (Fig. 2a, c) and *mcrA* genes and transcripts (Fig. 2b, d) showed an M-like up and down over incubation time. The copy numbers of bacterial 16S rRNA genes and transcripts peaked first on day 21 (1.2 × 10^10^ genes vs. 4.8 × 10^11^ transcripts g^-1^ dry soil) and again on day 35 (1.2 × 10^10^ genes vs. 4.2 × 10^11^ transcripts g^-1^ dry soil), with an intermediate decrease on day 28 (5.7 × 10^9^ 16S rRNA genes vs. 2.7 × 10^11^ transcripts g^-1^ dry soil) (Fig. 2a,c).

Likewise, the copy numbers of *mcrA* genes and transcripts peaked first on day 21 (2.9 × 10^9^ *mcrA* genes vs. 9.2 × 10^10^ transcripts g^-1^ dry soil) and again on day 35 (3.4 × 10^9^ *mcrA* genes vs. 6.5 × 10^10^ transcripts g^-1^ dry soil), with an intermediate decrease on day 28 (1.3 × 10^9^ *mcrA* genes vs. 3.8 × 10^10^ transcripts g^-1^ dry soil) (Fig. 2b, d).

After day 35, the gene and transcript copy numbers of both bacterial 16S rRNA and *mcrA* strongly decreased towards day 120 (Fig. 2).

### The bacterial metatranscriptome (16S rRNA, mRNA)

#### Taxon-specific dynamics

**Fig. 3:**
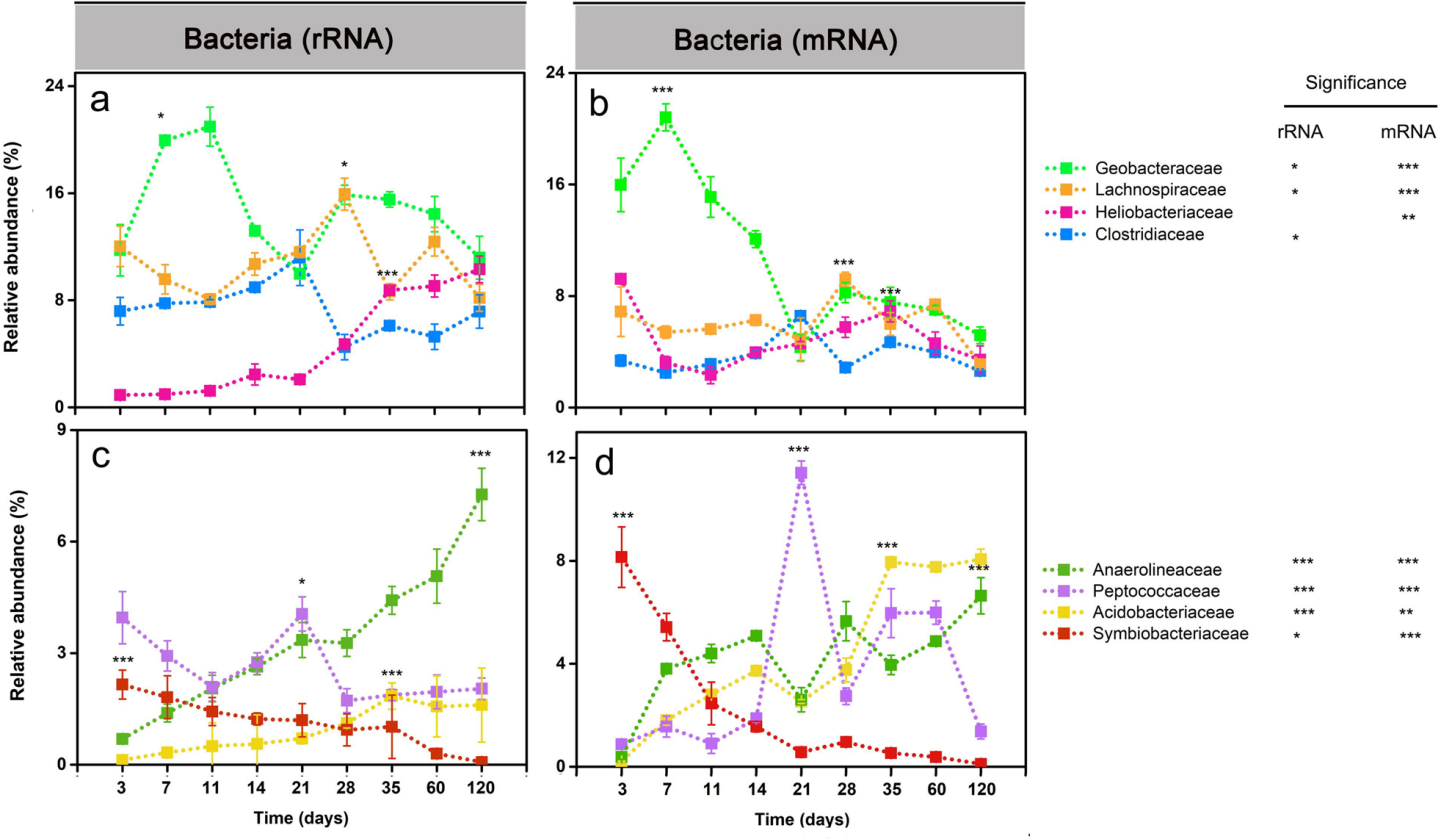
Relative abundance changes of dominant bacterial families (> 2%) on rRNA (**a**, **c**) and mRNA (**b**, **d**) levels, respectively. The percentage abundances are given in relation to total bacterial 16S rRNA and mRNA, respectively. Data are means ± SE (*n* = 3). Asterisks * (*P*_*FDR*_≤0.05), ** (*P*_*FDR*_≤0.01), and *** (*P*_*FDR*_≤0.001) indicate significant differences. Asterisks shown aside from the taxonomic names indicate that in STAMP, the relative rRNA or mRNA abundances significantly changed through the whole incubation period. Asterisks directly shown in the plots indicate that in DESeq2, the rRNA or mRNA abundance in that particular sampling time point significantly differed from the original abundance at the first sampling (day 3) or vice versa that the relative rRNA or mRNA abundance at day 3 (*Symbiobacteriaceae*) significantly differed from all other sampling time points. Significance values (*P*_*FDR*_) are only indicated on rRNA and mRNA levels for the taxon-specific abundance peak. The complete set of *P*_*FDR*_ values obtained by DESeq2 analysis for all sampling time points is shown in Tables S10 (rRNA) and S11 (mRNA)

The family-level population dynamics varied over incubation time (Figs. 3 and S3, Tables S10 and S11). At the rRNA level, the relative abundance of *Geobacteraceae* peaked between days 7 and 11 (Fig. 3a). Thereafter, the abundance of *Clostridiaceae and Peptococcaceae* peaked at day 21, while *Lachnospiraceae* showed the maximum abundance on day 28 (Fig. 3a,c). In the late phase (days 35 to 120), *Heliobacteriaceae* and *Anaerolineaceae* showed increased rRNA abundances and were, together with *Geobacteraceae*, the predominant bacterial populations (Fig. 3a,c).

Like on the 16S rRNA level, the mRNA abundance of the *Geobacteraceae* peaked on day 7, covering 22% of total community-wide mRNA and 36% of total bacterial mRNA. This was followed by a rapid abundance decline towards day 21 and another increase thereafter (Figs. 3b and S4). The *Peptococcaceae* reached their peak mRNA abundance on day 21 (Fig. 3d). Likewise, *Clostridiaceae* (day 21) and *Lachnospiraceae* (day 28) showed peak mRNA abundances consistent with those observed on the rRNA level. The *Acidobacteriaceae* and *Anaerolineaceae* displayed higher mRNA abundances in the late phase (days 35 to 120) than in the early phase (days 3 to 21) (Fig. 3d)*.* In addition, putative syntrophic populations, such as *Peptococcaceae* and *Heliobacteriaceae*, reached a second peak abundance during the late phase (Fig. 3b, d).

#### Carbohydrate-active enzymes (CAZymes)

A total of 13,632 mRNA contigs were annotated to encode CAZymes involved in degrading cellulose (e.g., cellulases, cellulose-1,4-beta-cellobiosidase), xylan (e.g., endo-1,4-beta-xylanase), other hemicelluloses (e.g., alpha- and beta-glactosidase), and chitin (chitinases) (Fig. S5a and Table S12). The majority of CAZyme transcripts were taxonomically assigned to *Firmicutes*, *Proteobacteria*, and *Actinobacteria*. Except for chitinases, CAZyme transcripts affiliated with *Firmicutes* steadily decreased in abundance with incubation time, while those affiliated with *Planctomycetes* increased. The abundance of CAZyme transcripts affiliated with *Proteobacteria* varied over incubation time. The abundance of CAZyme transcripts affiliated with *Actinobacteria* was particularly high during the final incubation period (day 120), except for CAZyme transcripts involved in degrading other hemicellulases (Fig. S5b and Table S12).

### The methanogen metatranscriptome (16S rRNA, mRNA)

**Fig. 4:**
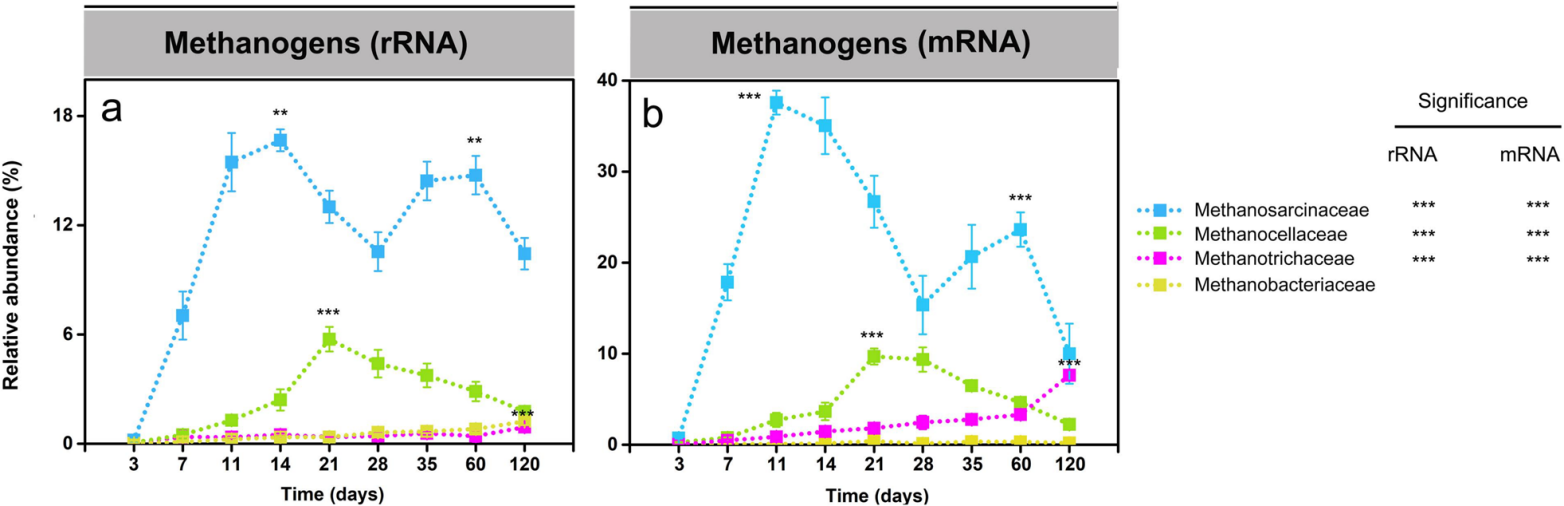
Relative abundance changes of the dominant methanogenic families (> 2%) on rRNA (**a**) and mRNA (**b**) levels, respectively. Their percentage abundances are given in relation to total archaeal 16S rRNA and mRNA, respectively. Data are means ± SE (*n* = 3). Asterisks * (*P*_*FDR*_≤0.05), ** (*P*_*FDR*_≤0.01), and *** (*P*_*FDR*_≤0.001) indicate significant differences. Asterisks shown aside from the taxonomic names indicate that in STAMP, the relative rRNA or mRNA abundances significantly changed through the whole incubation period. Asterisks directly shown in the plots indicate that in DESeq2, the rRNA or mRNA abundance in that particular sampling time point significantly differed from the original abundance at the first sampling (day 3). Significance values (*P*_*FDR*_) are only indicated on rRNA and mRNA levels for the taxon-specific abundance peak. The complete set of *P*_*FDR*_ values obtained by DESeq2 analysis for all sampling time points is shown in Tables S10 (rRNA) and S11 (mRNA)

*Methanosarcinaceae*, *Methanocellaceae*, and *Methanotrichaceae* were the prevailing methanogenic families throughout the complete incubation period (Fig. 4a, b; Tables S10 and S11). The *Methanosarcinaceae* was the dominant methanogen group, showing two-peak abundance dynamics. The family-level rRNA and mRNA abundances peaked first around days 11 and 14, then decreased until day 28, but increased thereafter again and reached a second activity peak between days 35 and 60. The *Methanocellaceae* were first detectable on day 11. Following the peak abundance on day 21, the relative rRNA and mRNA abundances steadily decreased towards day 120. Significant transcript levels of the *Methanotrichaceae* were only detectable on mRNA level during the late phase, with the peak abundance at day 120.

#### Defining the dominant Methanosarcina populations

**Fig. 5:**
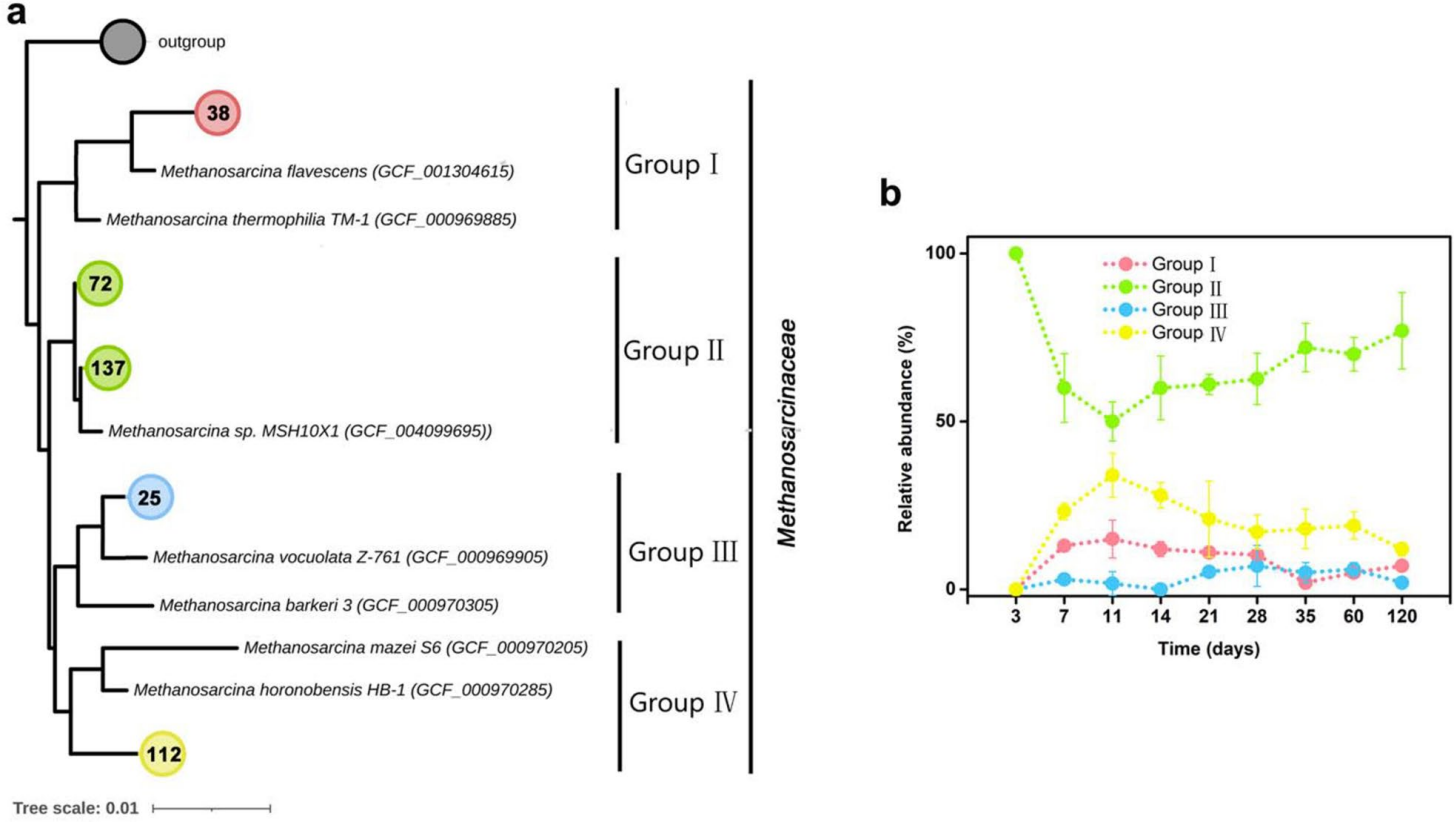
Phylogenetic tree of the four *Methanosarcina* populations detected in the paddy soil slurries (**a**) and their relative abundance dynamics over incubation time (**b**). **a** The neighbor-joining tree was constructed based on near full-length 16S rRNA sequences (> 1200 bp) assembled by EMIRGE from the metatranscriptomic datasets (415 sequences) and reference sequences extracted from *Methanosarcina* genomes in GTDB. **b** The relative abundance dynamics of the four distinct *Methanosarcina* populations (Groups I to IV) were inferred from the assembled 16S rRNA sequences. Data are means ± SE (*n* = 3)

The assembled near-full-length 16S rRNA sequences grouped into four distinct *Methanosarcina* populations (Groups I to IV) (Fig. 5a). These differed in their abundance dynamics (Fig. 5b). The Group II population was most closely related to *Methanosarcina* sp. MSH10X1 and predominant throughout slurry incubation. *Methanosarcina* Group IV was the second most abundant population and closely affiliated with *M*. *horanabensis*. The Group II and Group IV populations showed opposite 16S rRNA abundance dynamics, with both Group II minimum abundance and Group IV maximum abundance at day 11 (51% [Group II] versus 34% [Group IV] of total *Methanosarcina* 16S rRNA) (Fig. 5b). The *Methanosarcina* Group I and Group III populations displayed low but relatively stable transcript abundances (collectively < 20% of total *Methanosarcina* 16S rRNA) throughout slurry incubation (Fig. 5b).

#### Mapping-independent expression analysis of methanogenic pathways

**Fig. 6:**
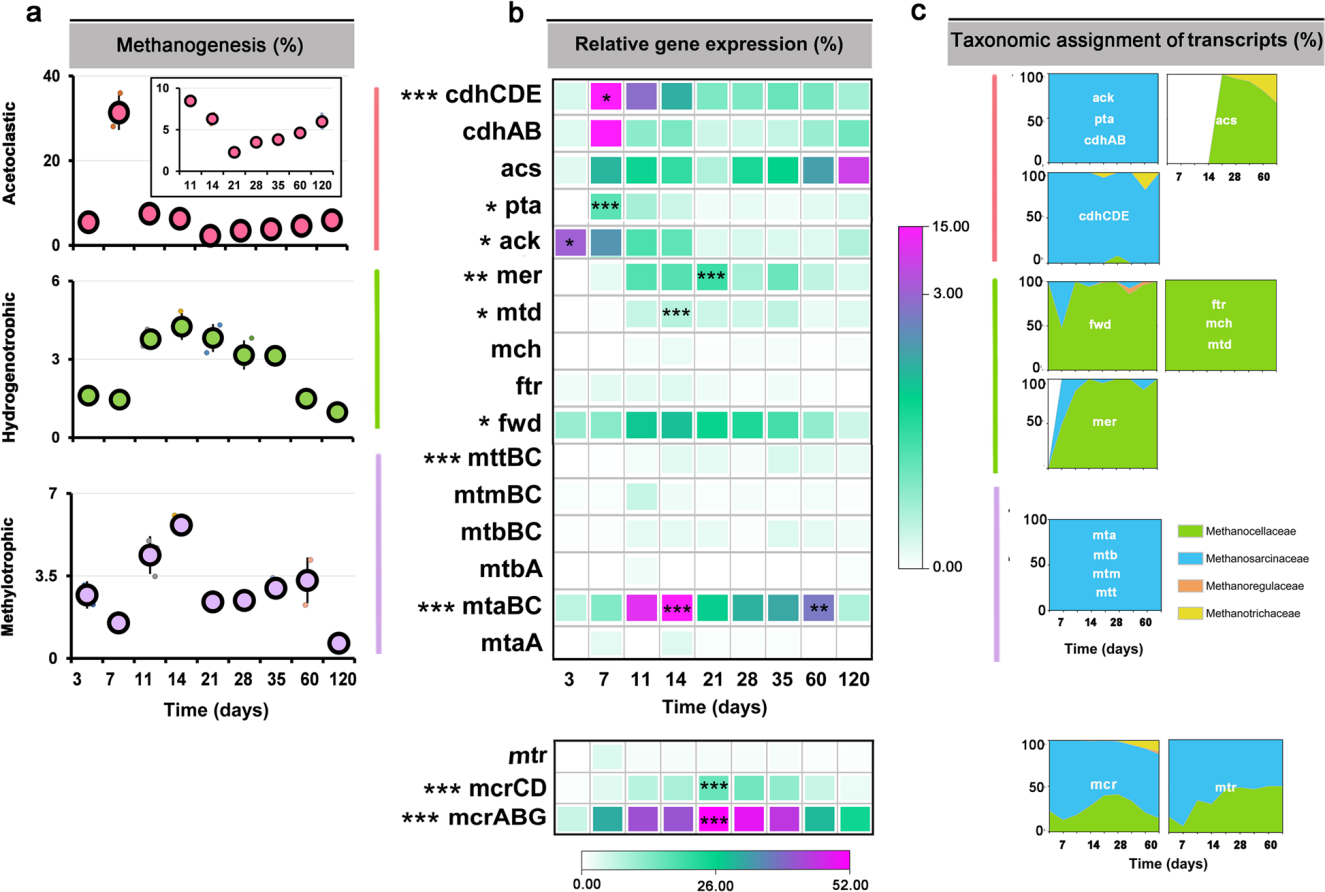
Relative mRNA abundance dynamics of individual methanogenic pathways over incubation time **a**, transcript dynamics of related key pathway genes **b**, and taxonomic assignment of the transcripts **c**. A list of the full gene names is shown in Table S14. The relative abundance values are given in relation to total mRNA affiliated to the KEGG level 3 category “methane metabolism”. The relative expression levels were calculated based on TPM values. Data are means ± SE (*n* = 3). Asterisks * (*P*_*FDR*_≤0.05), ** (*P*_*FDR*_≤0.01), and *** (*P*_*FDR*_≤0.001) indicate significant differences. Asterisks shown aside from the gene names indicate that in STAMP, the relative mRNA abundance significantly changed through the whole incubation period. Asterisks directly shown in the heatmap (**b**) indicate that in DESeq2, the mRNA abundance in that particular sampling time point significantly differed from the original abundance at the first sampling (day 3) or vice versa that the relative mRNA abundance (*ack*) at day 3 significantly differed from all other sampling time points. Significance values (*P*_*FDR*_) are only indicated for the peak transcript abundance(s) of each key pathway gene. **c** The percentage of *acs* transcripts affiliated with *Methanocellaceae* (acetate assimilation) was 68.1% on day 120, while those affiliated with *Methanotrichaceae* (acetoclastic methanogenesis) contributed 31.9%. The complete set of *P*_*FDR*_ values obtained in DESeq2 analysis for all sampling time points is shown in Table S13

The collective mRNA abundance of major methanogenesis pathways (Fig. 6a and Table S13) and the transcript abundance of particular pathway marker genes (Fig. 6b and Tables S13, S14) varied with incubation time. Acetoclastic methanogenesis was the dominant methane production pathway with the greatest transcript abundance in the early phase (36.4% of total mRNA assigned to KEGG level 3 category “methane metabolism”). This involved the peak transcript abundance of the following pathway marker genes: c*dhAB*, *cdhCDE*, *ack*, and *pta* (Fig. 6b). The changes in their relative expression level agreed well with the overall mRNA abundance dynamics of the *Methanosarcinaceae* during the early phase (Figs. 4b, 6 a-c). Transcripts of genes (*acs*) indicative of *Methanotrichaceae*-driven acetoclastic methanogenesis steadily increased in relative abundance during the late phase. Their peak abundance occurred on day 120, at which the relative expression level of genes indicative of *Methanosarcinaceae*-driven acetoclastic methanogenesis had significantly declined (Figs. 4b and 6b, c).

Members of the *Methanocellaceae* were the prevailing H_2_/CO_2_-utilizing methanogens, with genes encoding hydrogenotrophic methanogenesis (*fwd*, *ftr*, *mch*, *mtd*, *mer*) being most expressed by this family-level group (Fig. 6b, c). Their gene expression dynamics agreed well with the overall mRNA abundance dynamics of the *Methanocellaceae* (Figs. 4b and 6a, b).

In addition, transcripts of genes (*mtaA*, *mtaBC*, *mtbA*, *mtbBC*, *mtmBC*, *mttBC*) indicative of methylotrophic methanogenesis were detected, with *mtaBC* exhibiting the greatest transcript abundance throughout slurry incubation. The relative abundance of transcripts involved in methylotrophic methanogenesis increased from day 3 onwards and reached the first peak abundance (5.8% of total mRNA assigned to KEGG level 3 category “methane metabolism”) on day 14, but then decreased towards day 21 (2.1%). Thereafter, the relative transcript level of methylotrophic methanogenesis increased again and reached a second peak (3.4% of total mRNA assigned to KEGG level 3 category “methane metabolism”) around day 60 (Fig. 6a, b). This two-peak (14 and 60 days) abundance pattern of mRNA encoding methylotrophic methanogenesis agreed well with the overall abundance dynamics of *Methanosarcina*-affiliated rRNA (Fig. 4a) and mRNA (Fig. 4b), in both early and late phases. Indeed, the taxonomic assignment of the transcripts involved in methylotrophic methanogenesis showed that they were entirely expressed by the *Methanosarcinaceae* (Fig. 6c).

### Gene expression of methanogenic pathways by Methanosarcina Group II

#### Methanosarcina MAGs

Metagenomic sequencing yielded 54,672,109 reads after quality control (Table S5). The assembly of quality-filtered reads generated 781,362 contigs greater than 1000 bp and 21,770 contigs greater than 5000 bp. This resulted in three medium-quality *Methanosarcina* MAGs, ranging in completeness from 62 to 82.5% (Table S5). One each was obtained from slurry material sampled after 21, 28, and 35 days of incubation. The three MAGs shared high average nucleotide identity (ANI) values (> 95%). Compared to *Methanosarcina* genomes in GTDB, the three MAGs shared the greatest ANI values with *Methanosarcina* sp. MSH10X1 (> 85%) (Fig. S6a). In addition, the three MAGs shared high nucleotide sequence identities of their *mcrA* genes (98%). When compared to *Methanosarcina* reference genomes, the three MAGs also shared the greatest *mcrA* nucleotide sequence identity values with *Methanosarcina* sp. MSH10X1 (> 94%) (Fig. S6b). The inclusion of a near full-length 16S rRNA gene sequence (1400 nt) from MAG_21 in the *Methanosarcina* 16S rRNA tree confirmed that the three MAGs belong to *Methanosarcina* Group II and are most closely related to *Methanosarcina* sp. MSH10X1 (Fig. S7). Their predicted genome size (3.42 Mbp) was highly similar to that of strain MSH10X1 (3.56 Mbp) (Fig. S8). Comparative genomics revealed that the three MAGs share the majority of functionally annotated genes with the reference genomes of *Methanosarcina* Groups I to IV (922 common genes), while they encode 141 unique genes (Fig. S9 and Table S15). A synteny analysis showed that the organization of the *mtaABC* genes encoding methanol:CoM methyltransferase/methanol corrinoid protein displays in the three *Methanosarcina* MAGs a higher similarity to that in *Methanosarcina* sp. MSH10X1 than to their organization in the other reference genomes (Fig. S10). The three *Methanosarcina* MAGs and the genome of strain MSH10X1 contained five gene copies of *mtaA* and three gene copies of *mtaBC* (Fig. S10 and Table S16)*.*

#### Mapping-dependent gene expression analysis

**Fig. 7:**
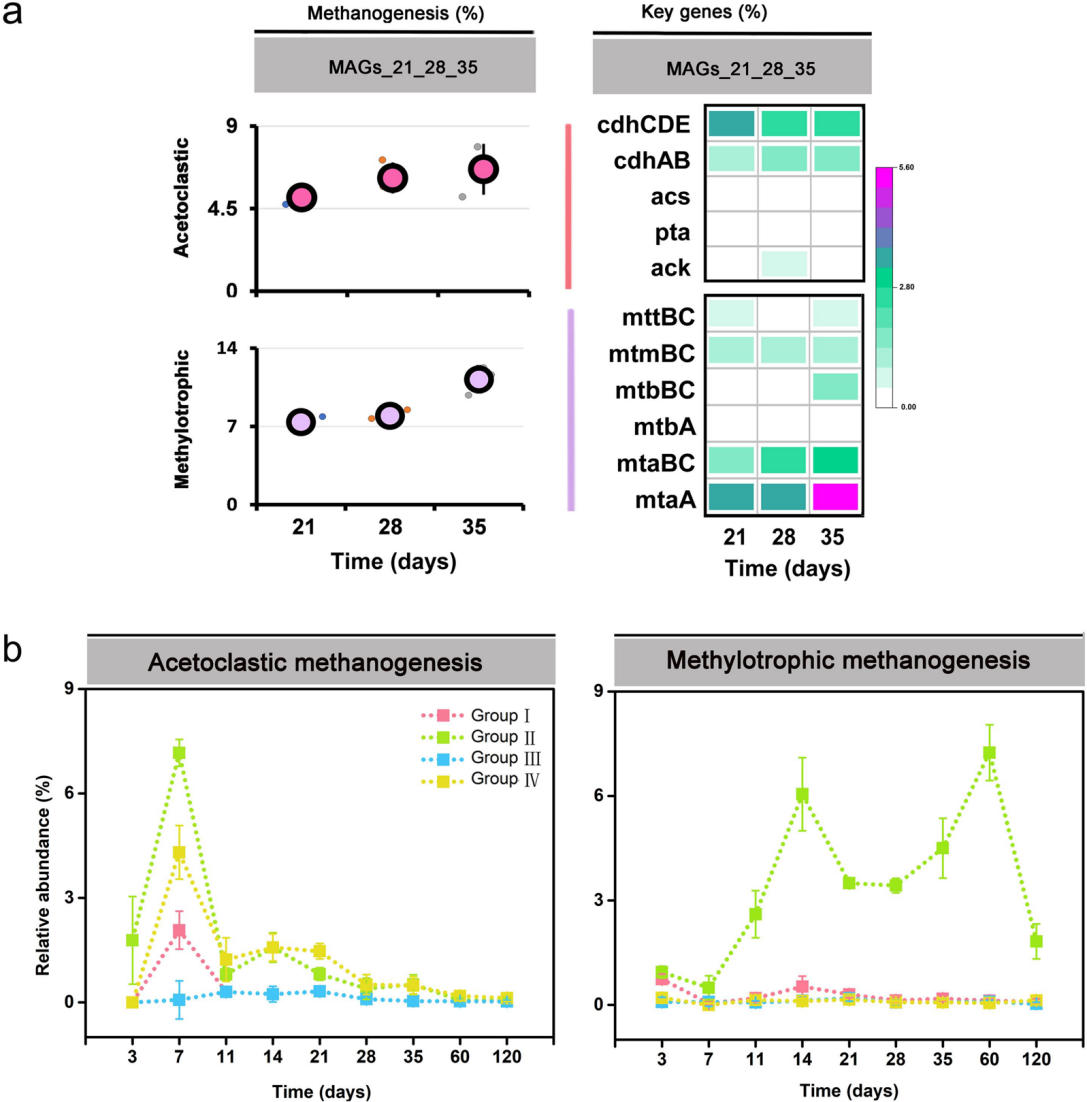
**a** Relative abundance of pathway-specific mRNA and transcripts of key pathway genes in relation to total mRNA that could be mapped onto the *Methanosarcina* MAGs. A list of the full gene names is shown in Table S14. Mapping onto the MAGs was conducted for each mRNA dataset retrieved in triplicate from the same incubation time point (days 21, 28, and 35). The small dots indicate the mapping efficiency of each mRNA replicate dataset, while the large dot represents the mapping efficiency averaged across all three mRNA replicate datasets of a given sampling time point. The complete set of *P*_*FDR*_ values obtained in DESeq2 analysis for all sampling time points is shown in Table S19. **b** Relative transcript dynamics of acetoclastic and methylotrophic methanogen pathways inferred from the competitive transcript mapping across the mix of *Methanosarcina* Group I to IV reference genomes. The relative mapping efficiencies were calculated based on TPM values. Data are means ± SE (*n* = 3). The mapping statistics are shown in Table S20

KEGG-annotated transcripts were mapped onto the three MAGs (Figs. 7a and S11). On KEGG level 2, transcripts involved in energy metabolism showed the greatest mapping efficiency (54% to 86% of total mapped mRNA) throughout slurry incubation, while transcripts encoding the translational apparatus were mapped with high frequency (30–32% of total mapped mRNA), particularly during the first week of slurry incubation (Fig. S11a and Table S17). On KEGG level 3, transcripts involved in methane metabolism were dominantly mapped (51 to 80% of total mapped mRNA) onto the MAGs throughout slurry incubation (Fig. S11b and Table S18). On KEGG level 4, transcripts involved in acetoclastic methanogenesis and methylotrophic methanogenesis were mapped with high frequency (Fig. 7a and Table S19). Overall, the transcript mapping frequency of the methylotrophic pathway (5.1–7.2% of total mapped mRNA) was lower than that of the acetoclastic pathway (7.5–12.4% of total mapped mRNA). Among total methanogen mRNA, *mtaABC* transcripts showed the greatest mapping frequency (Fig. 7a).

### Competitive transcript mapping onto the four reference genomes

Competitive mapping of metatranscriptomic mRNA reads onto the composite reference genomes of *Methanosarcina* Groups I to IV revealed that the vast majority of transcripts involved in acetoclastic and methylotrophic methanogenesis were mapped onto the genome of *Methanosarcina* sp. MSH10X1 (Group II). The only exception was metatranscriptomic reads obtained from the paddy soil slurries after 7-day incubation. On that day, a certain number of transcripts involved in acetoclastic methanogenesis were also mapped onto the genomes of *Methanosarcina flavescens* (Group I) and *Methanosarcina horonobensis* (Group IV) (Fig. 7b and Table S20).

## Discussion

Our study revealed successional dynamics of distinct methanogen populations over the 120-day incubation period. This was closely linked to changes in the expression level of major methanogenesis pathways, which will be discussed in greater detail in the following text. A discussion on the dynamics of the bacterial community is made in supplementary text (Additional File 3).

### Linking metabolite turnover with methanogen dynamics

Typical intermediates (i.e., acetate, propionate, butyrate) of the methanogenic food web transiently accumulated and were then rapidly consumed. Acetate is the most abundant intermediate during the anaerobic organic matter breakdown [19, 70]. Generally, it is the direct substrate for acetoclastic methanogens, including *Methanosarcinaceae* and *Methanotrichaceae* [70–72]. The transient accumulation of acetate with highest peak concentration on day 7 followed by its fast methanogenic consumption was closely linked to the increase in both the activity (defined by increased mRNA abundance) of *Methanosarcinaceae* (days 7 to 14, Fig. 4b) and the CH_4_ production rate (days 11-21, Fig. 1d). Thereafter, the decline in *Methanosarcinaceae*’s activity (days 14 to 28, Fig. 4b) corresponded well to a transient decrease in CH_4_ production (days 21 to 28, Fig. 1d).

Propionate and butyrate are the next important intermediates of organic matter conversion under anoxic conditions [73]. Acetate, H_2_, and CO_2_ generated by the syntrophic conversion of propionate and butyrate can feed acetoclastic and hydrogenotrophic methanogens. The oxidation of propionate (*G*_0_' = +76 kJ/mol) and butyrate (G_0_' = +48,3 kJ/mol) is highly endergonic under standard conditions but can be accomplished by syntrophy with an H_2_-utilizing methanogen, which maintains a low H_2_ partial pressure [74]. The rapid consumption of butyrate between days 7 and 11 (Fig. 1b) corresponds well to the initial activity increase of H_2_-utilizing *Methanocellaceae* (Fig. 4b). The net consumption of propionate from day 14 towards day 28 indicates that in addition to butyrate, propionate fueled acetoclastic methanogenesis (Fig. 1c).

### Composition and activity dynamics of the methanogenic community

Our study identified three successional phases defined by two activity peaks separated by an intermittent decrease of methanogenic activity (Fig. 4). Methanogenic archaea obtain energy for growth by converting C1 and C2 compounds, including CO_2_, formate, acetate, ethanol, methanol, and other methylated compounds, to methane [75–77]. Thus, the succession of methanogen guilds is due to changes in available substrates and their utilization for energy conservation. *Methanosarcinaceae* were the first methanogens to be stimulated in response to substrates (acetate) released by the anaerobic degradation of rice straw and may be considered early- or rapid-responding methanogens (Figs. 1a and 4b). With the ongoing anaerobic degradation of complex carbon, the activity of *Methanocellaceae* as intermediate responders was stimulated, primarily due to their role as syntrophic methanogen partners in the bacterial conversion of propionate to acetate, H_2_, and CO_2_ (Figs. 1c and 4b) [78–81].

*Methanosarcinaceae* and *Methanotrichaceae* are an excellent example of how two family-level groups compete for the same substrate. *Methanosarcinaceae* prevailed in acetoclastic methanogenesis at the high acetate concentrations during the early phase, but after acetate declined to a very low level, the late-responding *Methanotrichaceae* significantly increased in competitiveness and acetoclastic activity (Figs. 1a and 4b). The abundance dynamics of total mRNA affiliated to either *Methanosarcinaceae* or *Methanotrichaceae* agreed well with changes in the relative expression level of their pathway genes indicative of acetoclastic methanogenesis (Figs. 4b and 6b, c). With a higher maximum rate of acetate utilization and maximum growth rate (*Y* * *k*), a higher half-saturation coefficient (KS), and a higher yield coefficient compared with *Methanotrichaceae*, elevated acetate concentrations are favorable for growth and activity of *Methanosarcinaceae*. By contrast, members of the *Methanotrichaceae* are superior at low acetate concentrations due to the investment of energy to activate acetate, thereby leading to a lower *k* and *KS* [82–84]. Hence, our results are consistent with the concept that both substrate availability and substrate concentration (threshold concept) are key factors controlling the structure and function of methanogenic communities.

A transient activity decline around day 28 was observed in the absolute abundance of biomarkers, including both genes and transcripts of bacterial 16S rRNA and methanogenic *mcrA* (Fig. 2). This activity decline was also well evidenced by changes in the metatranscriptomic abundance of methanogen rRNA and mRNA (Fig. 4). Previous research has shown that over the first 4 weeks of straw decomposition, labile polymers are much faster hydrolytically released than recalcitrant compounds; with the latter having a longer residence time [70, 85]. The anaerobic decomposition of labile straw components, such as pectin and xylan, primarily occurs by a bacterial food web attached to rice straw [6, 36]. Exhaustion of the labile straw components leads not only to a change in the microbial colonization of rice straw but also to a transient decline in methanogenic precursors. In particular, hydrolytic and fermentative bacteria are increasingly detached from rice straw and released into the soil [6, 36]. In consequence, the transition from degrading the labile straw components to decomposing recalcitrant C compounds presumably leads to an overall decline in community activity around day 28 until critical sizes of functionally active populations and methanogenic precursor pools are re-established, thereby leading to a second activity peak during the late phase.

### Characterization of dominant Methanosarcina populations

The intra-family analysis of *Methanosarcinaceae* identified four distinct populations, with the Group II population prevailing over the complete 120-day incubation period (Fig. 5). This implies the same methanogen population with close affiliation to *Methanosarcina* sp. MSH10X1 dominated *Methanosarcinaceae* during the early and late activity phases (Figs. 4 and 5). An ANI value of 95% is considered species demarcation [65]. Thus, our three *Methanosarcina* MAGs recovered from slurry samples incubated for 21, 28, and 35 days presumably belong to the same species. Moreover, the high ANI value found between the three *Methanosarcina* MAGs and *Methanosarcina* sp. MSH10X1 (around 85%) suggests that the MAGs and strain MSH10X1 represent different but closely related species of the Group II population. (Fig. S6a). Phylogenetic analysis of the 16S rRNA gene located on the *Methanosarcina* MAG-21 and representative full-length 16S rRNA sequences assembled from our metatranscriptomic datasets further substantiate that the three *Methanosarcina* MAGs are closely affiliated to *Methanosarcina* sp. MSH10X1. This provides additional evidence for the dominant role of the *Methanosarcina* Group II population in the slurry incubations (Fig. S7).

### Expression of methanogenic pathways by Methanosarcina spp.

Previous paddy soil studies have demonstrated that acetoclastic methanogenesis is the major methane production pathway operated by members of the *Methanosarcinaceae* [16, 19, 28]. Indeed, we observed a high expression level of key genes (*ack*, *pta*, and *cdhA-E*) related to acetoclastic methanogenesis by *Methanosarcina* populations*.* The expression of *ack* and *pta* reached their peak abundance in the early phase, while their transcripts were detected only on a low level in the late phase. Obviously, acetoclastic methanogenesis was the major pathway operated by *Methanosarcina* spp. in the early phase (Fig. 6). The competitive mapping results further confirmed the major role of acetoclastic methanogenesis in *Methanosarcina* populations (including Groups I, II, and IV) during the early phase (Fig. 7b).

However, we also detected the expression of key genes encoding methylotrophic methanogenesis *(mta*, *mtb*, *mtm*, and *mtt*), but in particular transcripts of *mtaBC* (accounting for 0.8 to 5.2% of total mRNA affiliated to the KEGG level 3 category “methane metabolism”). The enzyme methyltransferase/methanol corrinoid protein encoded by *mtaBC* is a specific biomarker for methanol-dependent methanogenesis (Fig. 6b). The transcripts involved in methylotrophic methanogenesis were affiliated with *Methanosarcinaceae*. While their greatest expression level agreed largely with the two activity peaks of *Methanosarcinaceae*, the first peak abundance of methylotrophic mRNA showed a time shift relative to the peak transcript abundance of acetoclastic methanogenesis in the early phase (Figs. 7b and 4b). Thus, it is reasonable to conclude that *Methanosarcinaceae* produce CH_4_ via acetoclastic and methylotrophic methanogenesis in the Philippine paddy soil under anoxic conditions. The competitive mapping approach resulted in the mapping of nearly all methylotrophic mRNA to the Group II representatives, thereby implying that *Methanosarcina* Group II was the major player in methylotrophic methanogenesis (Fig. 7b). Detection of the genes encoding methylotrophic methanogenesis on the three *Methanosarcina* MAGs further substantiates this conclusion (Fig. 7a). Actually, *Methanosarcina* strain MSH10X1 prefers methanol and trimethylamine as substrates over acetate, H_2_/CO_2_, and other methylated compounds [86]. The utilization of methanol and trimethylamine resulted in the greatest methane yield produced by strain MSH10X1. This pure-culture-based finding agrees well with our result that *Methanosarcina* Group II is most competitive under conditions favoring methylotrophic methanogenesis. The expression dynamics of the acetoclastic and methylotrophic pathways by the Group II population strongly differed over the 120-day incubation period, thereby providing evidence that they were differentially regulated in response to major changes in substrate availability (Figs. 6 and 7b**)**. In addition to methanol released during pectin degradation, certain amounts of methanol released during the decomposition of xylan [87, 88] and lignin [89–91] may have fed methylotrophic methanogenesis. Recalcitrant lignin may have been the primary source of methanol for methylotrophic methanogenesis during the second expression peak around incubation day 60. Here, it is noteworthy that compared to other major rice farming areas (China, Italy), the Philippine rice field soil is particularly rich in humic acids [32]. The anaerobic decomposition of lignin and the release of methanol requires the activity of bacterial laccases-like enzymes that anaerobically depolymerize lignin into smaller polymer units to which the enzyme pool of the anaerobic bacteria can get access to decompose them into monomers [90, 91]. Among the bacterial community, members of the *Geobacteraceae* are the most promising candidates for being involved in the anaerobic lignin degradation. These bacteria showed not only the expression of putative laccase genes but also their greatest relative expression level during the later phase (days 28 and 35) (Table S21). Thus, a functional interplay may have occurred between members of the *Geobacteraceae* and the *Methanosarcina* Group II population, a view that is further supported by the correspondence of their abundance dynamics on both rRNA and mRNA levels (compare Figs. 3 and 4; and see Additional File 3 for further details). Another putative candidate for being involved in the degradation of recalcitrant lignin may be members of the *Peptococcaceae*, given the reasonable correspondence of their rRNA and mRNA peak abundances with the expression peaks of methylotrophic methanogenesis. The *Peptococcaceae* have been identified as putative aromatic ring-cleavage bacteria in rice field soil under lignin-degrading methanogenic conditions [92].

### Final remarks

Understanding the sources and controls of microbial methane production and the response mechanisms of methanogenic communities in rice field soils is critical for modeling predictions of global methane emissions. Metatranscriptomics, coupled in part with metagenomics, provided us with detailed insights into the role and dynamics of key functional guilds participating in the methanogenic organic matter breakdown in Philippine paddy soil. A particular *Methanosarcina* population closely related to strain MSH10X1 dominated the methanogen community over the complete incubation period. This species-level population showed a significant successional change in the methanogenic pathways operated during the incubation period, with acetoclastic methanogenesis being highly active during the very early phase (around day 7) and methylotrophic methanogenesis being active during the later stages involving two independent expression peaks after an incubation period of 14 and 60 days. Collectively, our research findings expand our hitherto knowledge of the methanogenic pathways being active in paddy soils and show that methylotrophy is a predominant methanogenesis pathway in certain rice field soils.

### Supplementary Information

**Additional file 1:** **Fig. S1.** Schematic diagram of the methanogenic food web and the three major methanogenesis (acetoclastic, hydrogenotrophic, and methylotrophic) pathways. A list of the full enzyme names is shown in Table S14. **Fig. S2.** Schematic presentation of the experimental design to investigate the methanogenic community dynamics in Philippine paddy soil. The slurries were incubated under anoxic conditions at 30 °C for 120 days. The research combined metabolite measurements (CH_4_, acetate, propionate, and butyrate), quantitative real-time PCR and RT-PCR of particular biomarkers (16S rRNA, *mcrA*), and meta-omics (environmental genomics and transcriptomics). **Fig. S3.** Metatranscriptomic abundance dynamics of bacterial and archaeal 16S rRNA (a) and mRNA (b) (cutoff > 2%) analyzed on family level over the 120-day incubation period. Taxonomic assignment of assembled 16S rRNA reads was performed using BLASTN algorithm implemented in DIAMOND against SILVA 132 SSU database with 0.90 sequence identity. Taxonomic assignment of mRNA reads was performed using BLASTX algorithm implemented in DIAMOND against NR database with 0.93 sequence identity. The relative abundance values are given in relation to total bacterial and archaeal metatranscriptomic 16S rRNA and mRNA. **Fig. S4. **Transcript dynamics of genes affiliated to the family *Geobacteraceae* and involved in either the synthesis of *c*-type cytochromes (a) or the KEGG category ‘cell motility’ (b). The relative abundance values are given in relation to total mRNA that could be functionally annotated in KEGG and is affiliated to the family *Geobacteraceae*.**Fig. S5.** Metatranscriptomic expression dynamics of genes encoding carbohydrate-active enzymes (CAZymes) that are involved in the breakdown of cellulose, xylan, other hemicelluloses, and chitin (a). Changes in the taxonomic composition of the CAZyme transcripts over the 120-day incubation period (b). The analysis involved multiple CAZyme families of glycosyl hydrolases (GHs) and carbohydrate-binding modules (CBMs). These CAZyme families are specified in Additional File 2, Table S12. **Fig. S6.** Average nucleotide identity (ANI) values calculated for the three *Methanosarcina* MAGs (21, 28, 35) and reference genomes downloaded from the Genome Taxonomic Database (GTDB) (a). Nucleotide sequence identities of the *mcrA* genes present in the same set of MAGs and *Methanosarcina* reference genomes (b). **Fig. S7.** Neighbor-joining tree showing the relationship between near full-length 16S rRNA sequences (> 1200 bp) assembled by EMIRGE from the metatranscriptomic datasets (415 sequences) and 16S rRNA gene sequences extracted from the complete *Methanosarcina* reference genomes downloaded from GTDB (a). Neighbor-joining tree showing the position of the near full-length 16S rRNA gene sequence of MAG_21 (1,400 nt) in relation to the 16S rRNA gene sequences extracted from the*Methanosarcina *reference genomes and near full-length 16S rRNA sequences (> 1200 bp) assembled by EMIRGE from the metatranscriptomic datasets. **Fig. S8.** Circular genome maps for MAG_35 (a) and the most closely related *Methanosarcina* reference genome (strain MSH10X1) (b). Circles from the outside to the inside show the positions of protein-coding sequences (blue), tRNA (red) and rRNA genes (green) on the positive (circle 1) and negative (circle 2) strands. Circle 3 shows the positions of BLAST hits detected through BLASTx (with an e-value cut-off of 1e-5) and circle 4 depicts the BLASTx results for the reciprocal search against the genome of strain MSH10X1 (a) and MAG_35 (b). Circles 5 and 6 show GC content and GC skew plotted as the deviation from the genomic average. **Fig. S9.** Venn diagram showing the distribution of functionally annotated genes among the three *Methanosarcina* MAGs (21, 28, 35) and the reference genomes of *Methanosarcina* Group I (*M. fluorescens*), Group II (strain MSH10X1), Group III (*M. barkeri*), and Group IV (*M. horonobensis*). The bar columns (pink) show the distribution pattern of particular sets of characterized genes among the MAGs and Group I to IV reference genomes. The green columns indicate the total number of predicted genes in the MAGs and Group I to IV reference genomes. The black and grey dots specify presence or absence of this particular set of characterized genes in the *Methanosarcina* MAGs and the Group I to Group IV reference genomes. Comparative genomics revealed that the three *Methanosarcina* MAGs (21, 28, 35) share their majority of predicted genes with the reference genomes of *Methanosarcina* Groups I to IV (922 characterized genes in common), while they encode 141 unique genes as specified in Table S15 (Additional File 2). The unique genes were associated with the following KEGG level 2 categories: cell wall component biosynthesis, cofactor biosynthesis, membrane transport, energy metabolism, cell motility, and genetic information processing. All the genes encoding essential enzymes for acetoclastic (*K00925*, *K00625*, *K00192*, *K00193*, *K00194*, *K00195* and *K00197*), hydrogenotrophic (*K00200-K00205*, *K11260*, *K11261*, *K00627*, *K01499*, *K00319* and *K00320*), and methanol-dependent methanogenesis *(K14080*, *K04480* and *K14081*) were identified on all three *Methanosarcina* MAGs as specified in Table S16 (Additional File 2). **Fig. S10.** Methanol-dependent methanogenesis pathway in the three *Methanosarcina* MAGs (21, 28, 35) and the *Methanosarcina* Groups I to IV reference genomes. *Methanosarcina *populations produce the MtaABC enzyme complex to catalyze the methyl transfer from methanol to CoM to form methyl-CoM (a and b). Comparison of the genetic organization of the *mta* gene cluster between the three *Methanosarcina* MAGs (21, 28, 35) and the Group I to IV reference genomes (c). **Fig. S11.** Relative abundances of mRNA mapped onto the three *Methanosarcina* MAGs (21, 28, 35) and affiliated with particular KEGG categories on level 2 (a) and 3 (b). The triplicate metatranscriptomes of a given incubation time point were individually mapped onto a particular MAG as follows: (i) triplicate metatranscriptomes generated from RNA of the sampling days 3, 7, 11, 14, and 21 onto MAG_21; (ii) triplicate metatranscriptomes generated from RNA of the sampling day 28 onto MAG_28; and (iii) triplicate metatranscriptomes generated from RNA of the sampling days 35, 60, and 120 onto MAG_35. The relative mapping efficiencies were calculated based on the normalized number of mRNA reads that could be mapped onto each ORF of a given *Methanosarcina* MAG. The dot sizes indicate relative mapping efficiencies. 

** Additional file 2:** **Table S1.** Forward and reverse primers used in qPCR and RT-qPCR. **Table S2.** Sequencing statistics of metatranscriptomic datasets. **Table S3.** Sequencing statistics of near full-length 16S rRNA sequences. **Table S4.** Sequencing statistics of mRNA sequences. **Table S5.** Sequencing statistics of the metagenomic datasets. **Table S6.** Significance analysis for the quantification of bacterial 16S rRNA gene copy numbers. **Table S7.** Significance analysis for the quantification of *mcrA* gene copy numbers. **Table S8.** Significance analysis for the quantification of bacterial 16S rRNA transcripts. T**able S9.** Significance analysis for the quantification of *mcrA *transcripts. **Table S10a.** Analysis of the taxon-specific abundance on rRNA level, using the software STAMP. *P*_*FDR*_ values ≤ 0.05 are indicative of signficant changes in family-level abundance across the complete 120-day incubation period. The resulting *p* values were corrected (*P*_*FDR*_) for multiple testing using the Benjamini-Hochberg method. **Table S10b.** Analysis for incubation time-dependent abundance changes of dominant family-level taxa on rRNA level, using the package DESeq2 in R. The resulting *p* values were corrected (*P*_*FDR*_) for multiple testing using the Benjamini-Hochberg method. *P*_*FDR*_ values ≤ 0.05 are indicative of signficant difference in taxon-specific rRNA abundance between two particular incubation time points. **Table S11a.** Analysis of the taxon-specific abundance on mRNA level, using the software STAMP. The resulting *p* values were corrected (*P*_*FDR*_) for multiple testing using the Benjamini-Hochberg method. *P*_*FDR*_ values ≤ 0.05 are indicative of signficant changes in family-level abundance across the complete 120-day incubation period. **Table S11b.** Analysis for incubation time-dependent abundance changes of dominant family-level taxa on mRNA level, using the package DESeq2 in R.The resulting *p* values were corrected (*P*_*FDR*_) for multiple testing using the Benjamini-Hochberg method. *P*_*FDR*_ values ≤ 0.05 are indicative of signficant difference in taxon-specific mRNA abundance between two particular incubation time points. **Table S12.** List of CAZyme families whose transcripts were detected in the metatranscriptomic datasets during the 120-day incubation period. **Table S13a.** Analysis for the mapping-independent abundance of genes involved in methanogenesis, using the software STAMP. The resulting *p* values were corrected (*P*_*FDR*_) for multiple testing using the Benjamini-Hochberg method. *P*_*FDR*_  values ≤ 0.05 are indicative of significant difference in relative transcript abundance across all the incubation times tested. **Table S13b.** Analysis for the mapping-independent abundance of genes involved in methanogenesis, using the package DESeq2 in R. The resulting *p* values were corrected (*P*_*FDR*_) for multiple testing using the Benjamini-Hochberg method. *P*_*FDR*_ values ≤ 0.05 are indicative of signficant difference in relative transcript abundance between two particular incubation time points. **Table S14.** The full name of enzymes involved acetoclastic, hydrogenotrophic, and  methylotrophic methanogenesis. **Table S15.** List of KEGG-annotated genes detected in the three *Methanosarcina* MAGs (21, 28, 35), but not in the *Methanosarcina* Groups I to IV reference genomes. **Table S16.** Copy number of KEGG-annotated genes present in both the three *Methanosarcina* MAGs (21, 28, 35) and the* Methanosarcina* Groups I to IV reference genomes. **Table S17.** KEGG level 2 analysis of mRNA mapped to the three *Methanosarcina* MAGs (21, 28, 35). The relative abundance values (%) are given in relation to total mapped mRNA functionally annotated by KEGG. The relative expression levels were calculated based on TPM values (means ± SE, *n* = 3). **Table S18.** KEGG level 3 analysis of mRNA mapped to the three *Methanosarcina* MAGs (21, 28, 35). The relative abundance values (%) are given in relation to total mapped mRNA functionally annotated by KEGG. The relative expression levels were calculated based on TPM values (means ± SE, *n* = 3). **Table S19a.** Analysis for the mapping-dependent abundance of genes involved in methanogenesis, using the software STAMP. The resulting *p* values were corrected (*P*_*FDR*_) for multiple testing using the Benjamini-Hochberg method. *P*_*FDR*_ values ≤ 0.05 are indicative of significant difference in transcript mapping accross all three MAGs. **Table S19b.** Analysis for the mapping-dependent abundance of genes involved in methanogenesis, using the package DESeq2 in R. The resulting *p* values were corrected (*P*_*FDR*_) for multiple testing using the Benjamini-Hochberg method. *P*_*FDR*_ values ≤ 0.05 are indicative of significant difference in transcript mapping between two particular incubation time points. **Table S20.** Statistics of transcript mapping to the three MAGs (21, 28, 25) and the *Methanosarcina* Groups I to IV reference genomes. **Table S21a.** Contigs of putative laccase-like genes (GeoLacc) expressed by members of the *Geobacteraceae*. **Table S21b.** TPM values of putative laccase-like genes (GeoLacc) expressed by members of the *Geobacteraceae*.

** Additional file 3:** Supplemental Discussion.

## Data Availability

The Illumina RNA-Seq data generated during this study have been deposited at the National Center for Biotechnology Information (NCBI) in the Sequence Read Archive under the BioProject accession number PRJNA907538. The metagenomic sequence data are accessible at NCBI under the BioProject accession number PRJNA907625.
