# Supplementary material for: Time-shifted expression of acetoclastic and methylotrophic methanogenesis by a single Methanosarcina genomospecies predominates the methanogen dynamics in Philippine rice field soil
Source: Microbiome. 2024 Feb 26;12:39. doi: 10.1186/s40168-023-01739-z (PMC10895765; doi:10.1186/s40168-023-01739-z)
Supplement: Supplementary file 2 — Additional file 1: Fig. S1. Schematic diagram of the methanogenic food web and the three major methanogenesis (acetoclastic, hydrogenotrophic, and methylotrophic) pathways. A list of the full enzyme names is shown in Table S14. Fig. S2. Schematic presentation of the experimental design to investigate the methanogenic community dynamics in Philippine paddy soil. The slurries were incubated under anoxic conditions at 30 °C for 120 days. The research combined metabolite measurements (CH4, acetate, propionate, and butyrate), quantitative real-time PCR and RT-PCR of particular biomarkers (16S rRNA, mcrA), and meta-omics (environmental genomics and transcriptomics). Fig. S3. Metatranscriptomic abundance dynamics of bacterial and archaeal 16S rRNA (a) and mRNA (b) (cutoff > 2%) analyzed on family level over the 120-day incubation period. Taxonomic assignment of assembled 16S rRNA reads was performed using BLASTN algorithm implemented in DIAMOND against SILVA 132 SSU database with 0.90 sequence identity. Taxonomic assignment of mRNA reads was performed using BLASTX algorithm implemented in DIAMOND against NR database with 0.93 sequence identity. The relative abundance values are given in relation to total bacterial and archaeal metatranscriptomic 16S rRNA and mRNA. Fig. S4. Transcript dynamics of genes affiliated to the family Geobacteraceae and involved in either the synthesis of c-type cytochromes (a) or the KEGG category ‘cell motility’ (b). The relative abundance values are given in relation to total mRNA that could be functionally annotated in KEGG and is affiliated to the family Geobacteraceae.Fig. S5. Metatranscriptomic expression dynamics of genes encoding carbohydrate-active enzymes (CAZymes) that are involved in the breakdown of cellulose, xylan, other hemicelluloses, and chitin (a). Changes in the taxonomic composition of the CAZyme transcripts over the 120-day incubation period (b). The analysis involved multiple CAZyme families of glycosyl hydrolases (GHs) and [file 40168_2023_1739_MOESM1_ESM.docx]

**Time-shifted expression of acetoclastic and methylotrophic methanogenesis by a single *Methanosarcina* genomospecies predominates the methanogen dynamics in Philippine rice field soil**

Xin Li^1#^, Qicheng Bei^1ω^, Mehrdad Rabiei Nematabad^1^, Jingjing Peng^2^,
and Werner Liesack^1*^

*^1^Research group “Methanotrophic Bacteria and Environmental Genomics/ Transcriptomics”, Max Planck Institute for Terrestrial Microbiology,*

*Karl-von-Frisch-Str. 10, Marburg, D-35043, Germany*

*^2^State Key Laboratory of Nutrient Use and Management, College of Resources and Environmental Sciences, National Academy of Agriculture Green Development,*

*Key Laboratory of Plant-Soil Interactions, China Agricultural University,
Beijing, 100193, China.*

**Supplemental Figures**

**Figures S1 to S11**

----

Supplemental Tables S1 to S21 are shown in Additional File 2

**
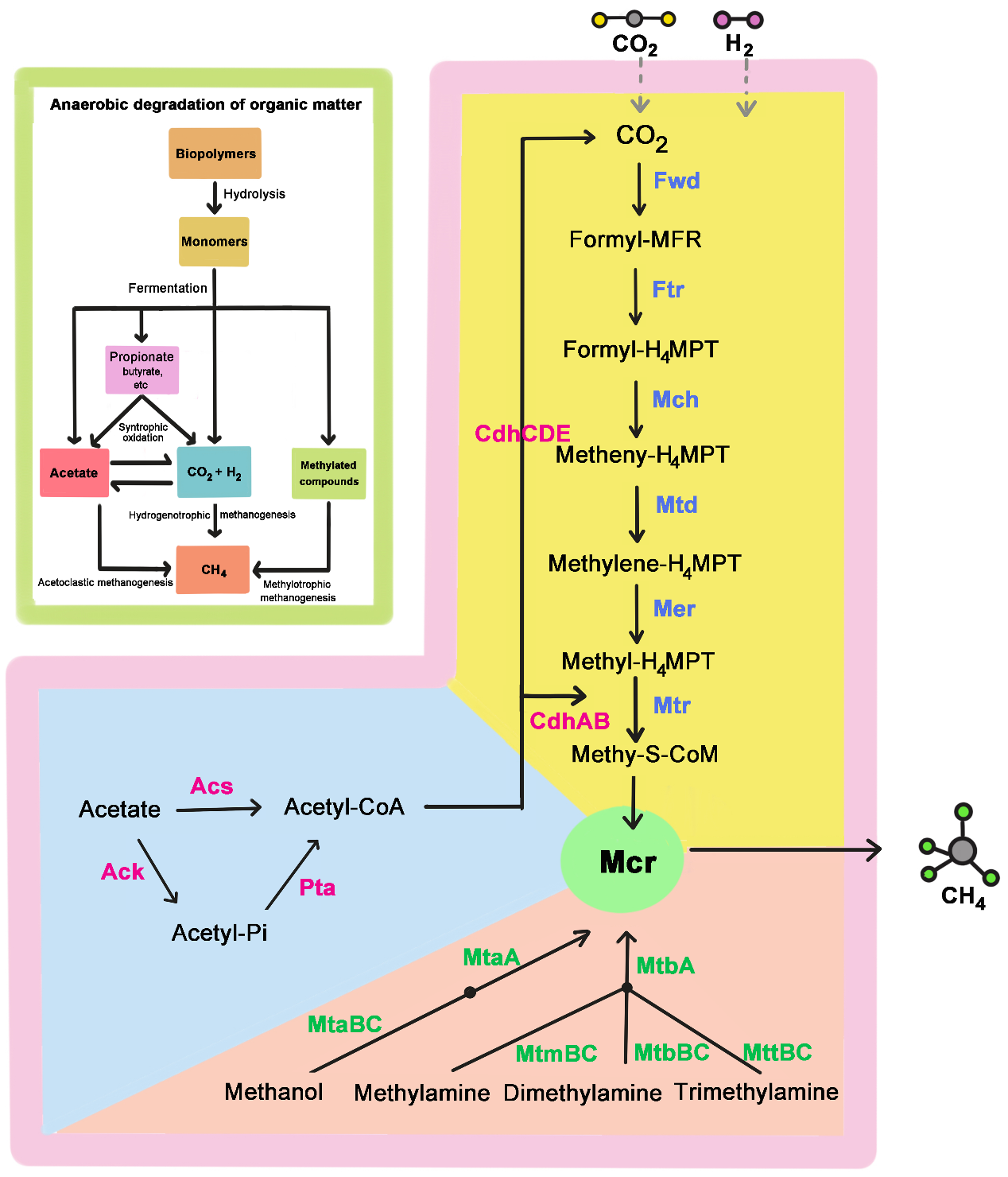
**

**Fig. S1** Schematic diagram of the methanogenic food web and the three major methanogenesis (acetoclastic, hydrogenotrophic, and methylotrophic) pathways. A list of the full enzyme names is shown in Table S14.


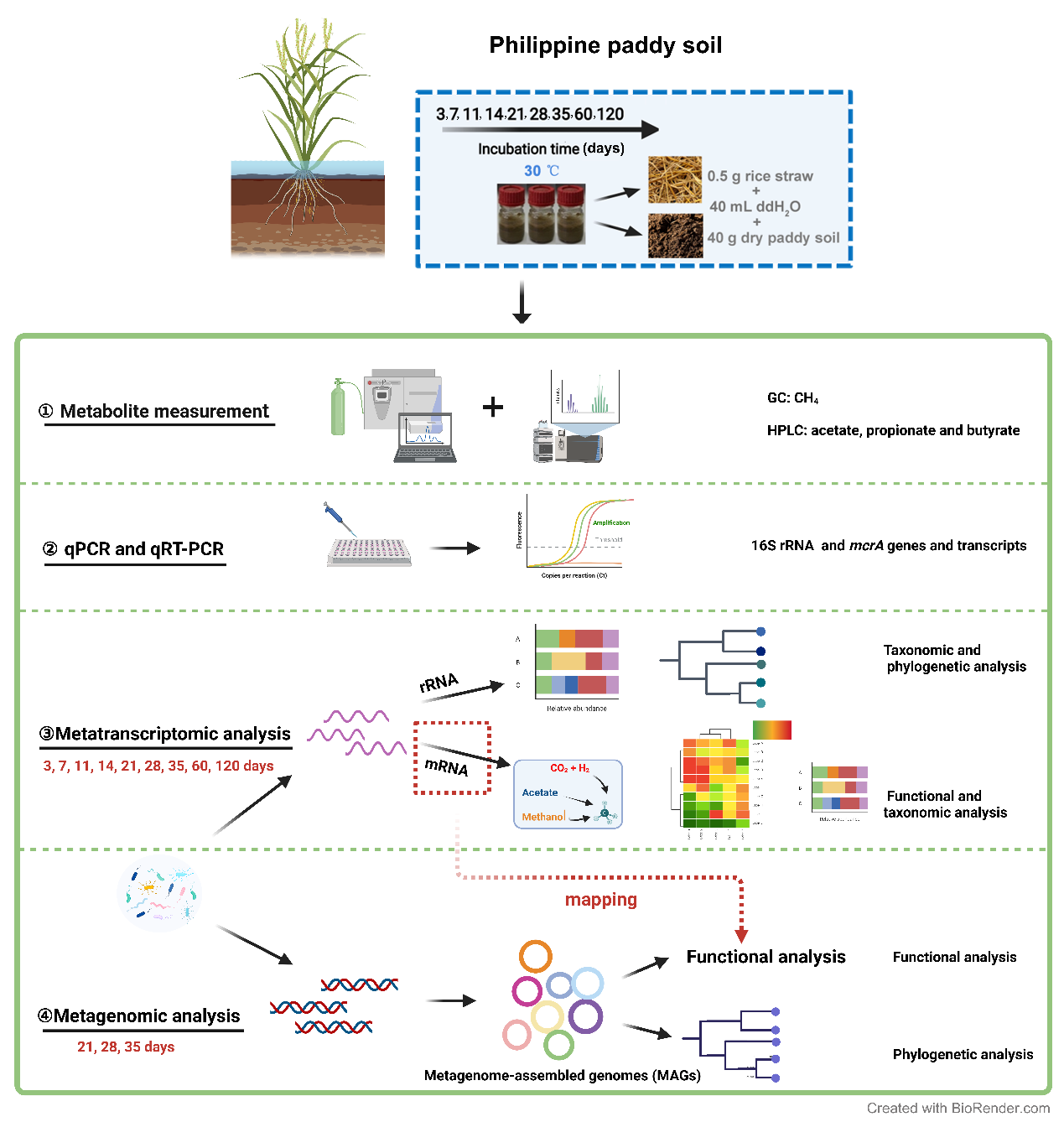


**Fig. S2** Schematic presentation of the experimental design to investigate the methanogenic community dynamics in Philippine paddy soil. The slurries were incubated under anoxic conditions at 30 °C for 120 days. The research combined metabolite measurements (CH_4_, acetate, propionate, and butyrate), quantitative real-time PCR and RT-PCR of particular biomarkers (16S rRNA, *mcrA*), and meta-omics (environmental genomics and transcriptomics).


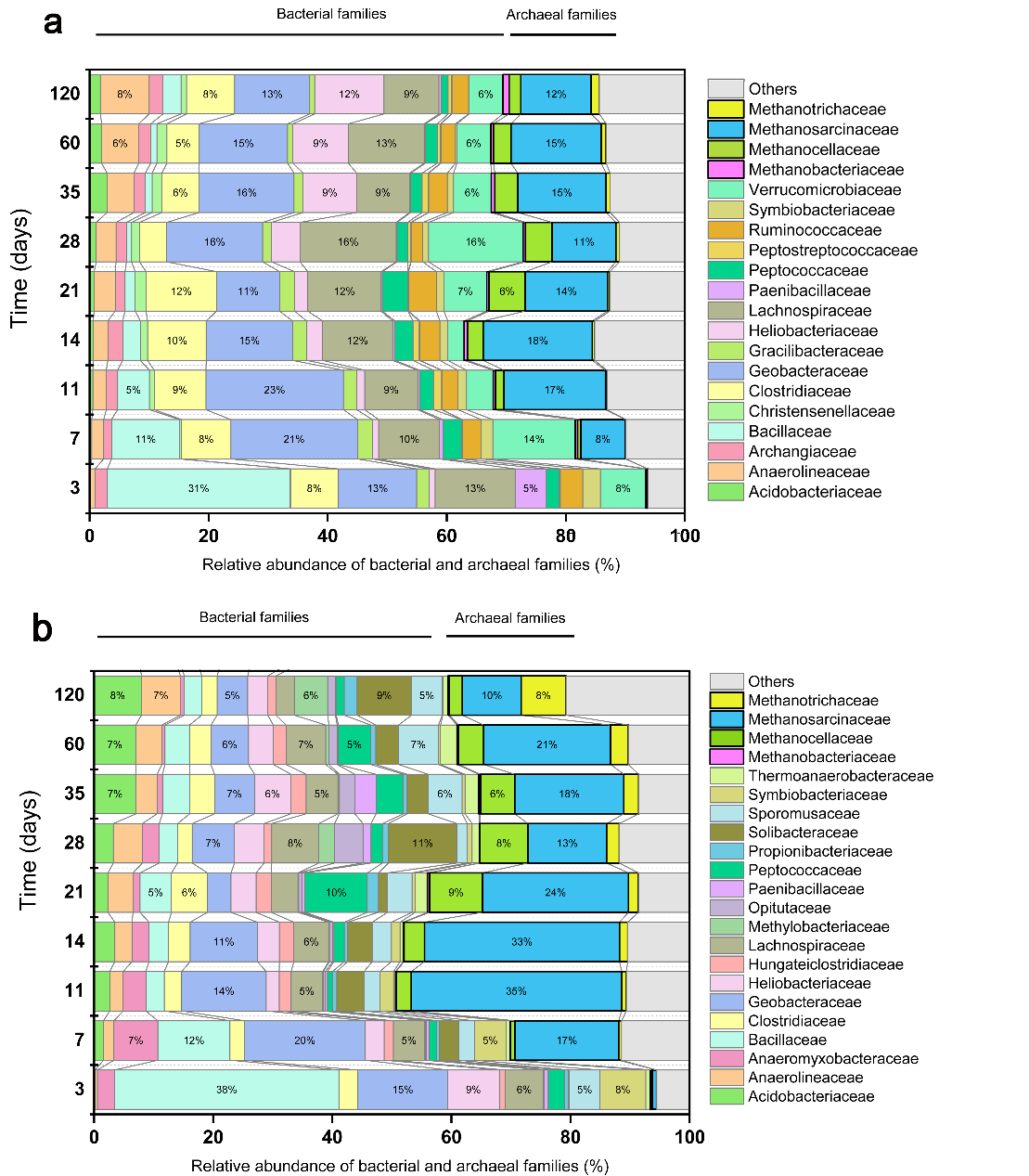


**Fig. S3** Metatranscriptomic abundance dynamics of bacterial and archaeal 16S rRNA (**a**) and mRNA (**b**) (cutoff > 2%) analyzed on family level over the 120-day incubation period. Taxonomic assignment of assembled 16S rRNA reads was performed using BLASTN algorithm implemented in DIAMOND against SILVA 132 SSU database with 0.90 sequence identity. Taxonomic assignment of mRNA reads was performed using BLASTX algorithm implemented in DIAMOND against NR database with 0.93 sequence identity. The relative abundance values are given in relation to total bacterial and archaeal metatranscriptomic 16S rRNA and mRNA.


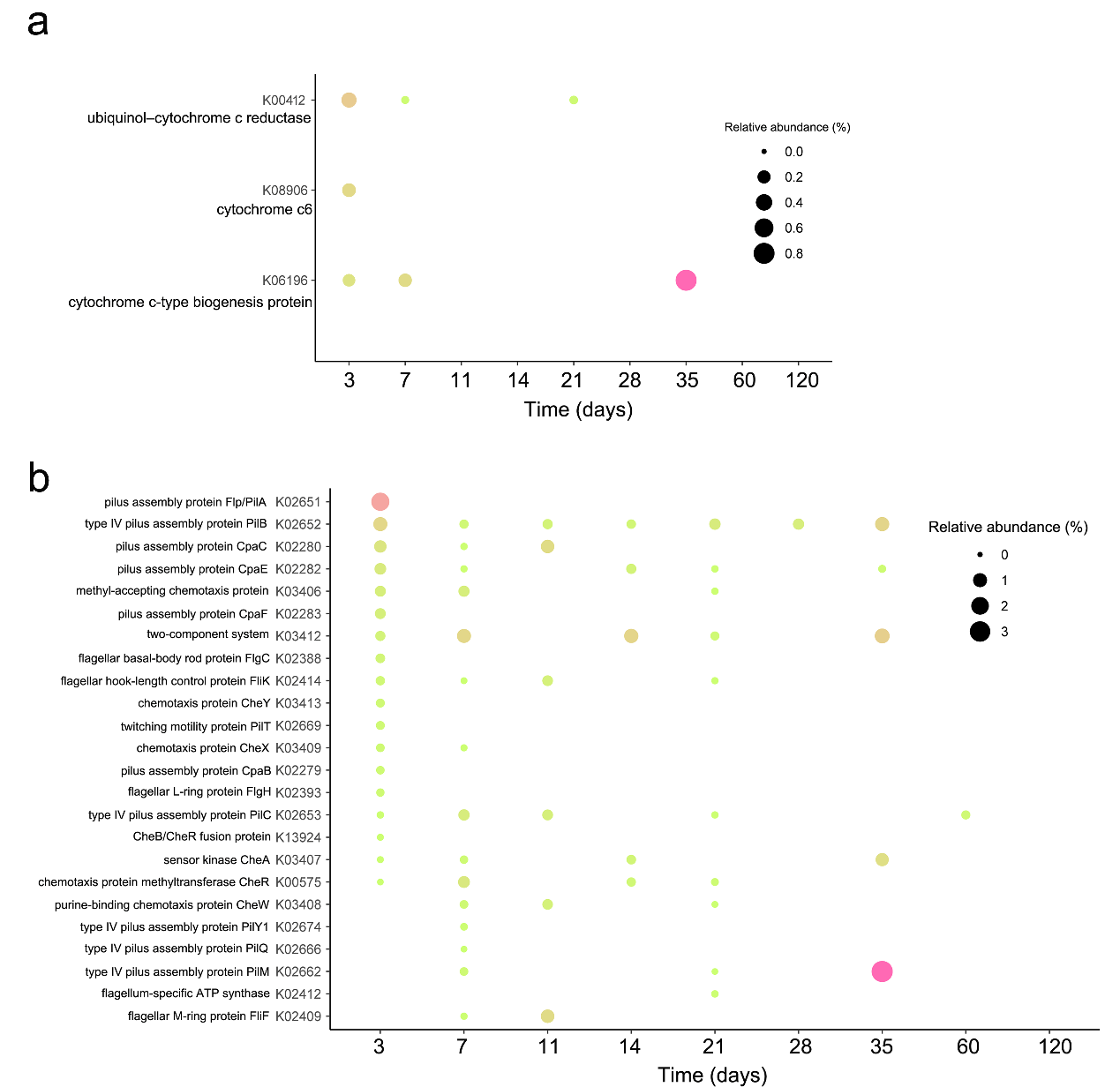


**Fig. S4** Transcript dynamics of genes affiliated to the family *Geobacteraceae* and involved in either the synthesis of *c*-type cytochromes (**a**) or the KEGG category ‘cell motility’ **(b)**. The relative abundance values are given in relation to total mRNA that could be functionally annotated in KEGG and is affiliated to the family *Geobacteraceae*.

_
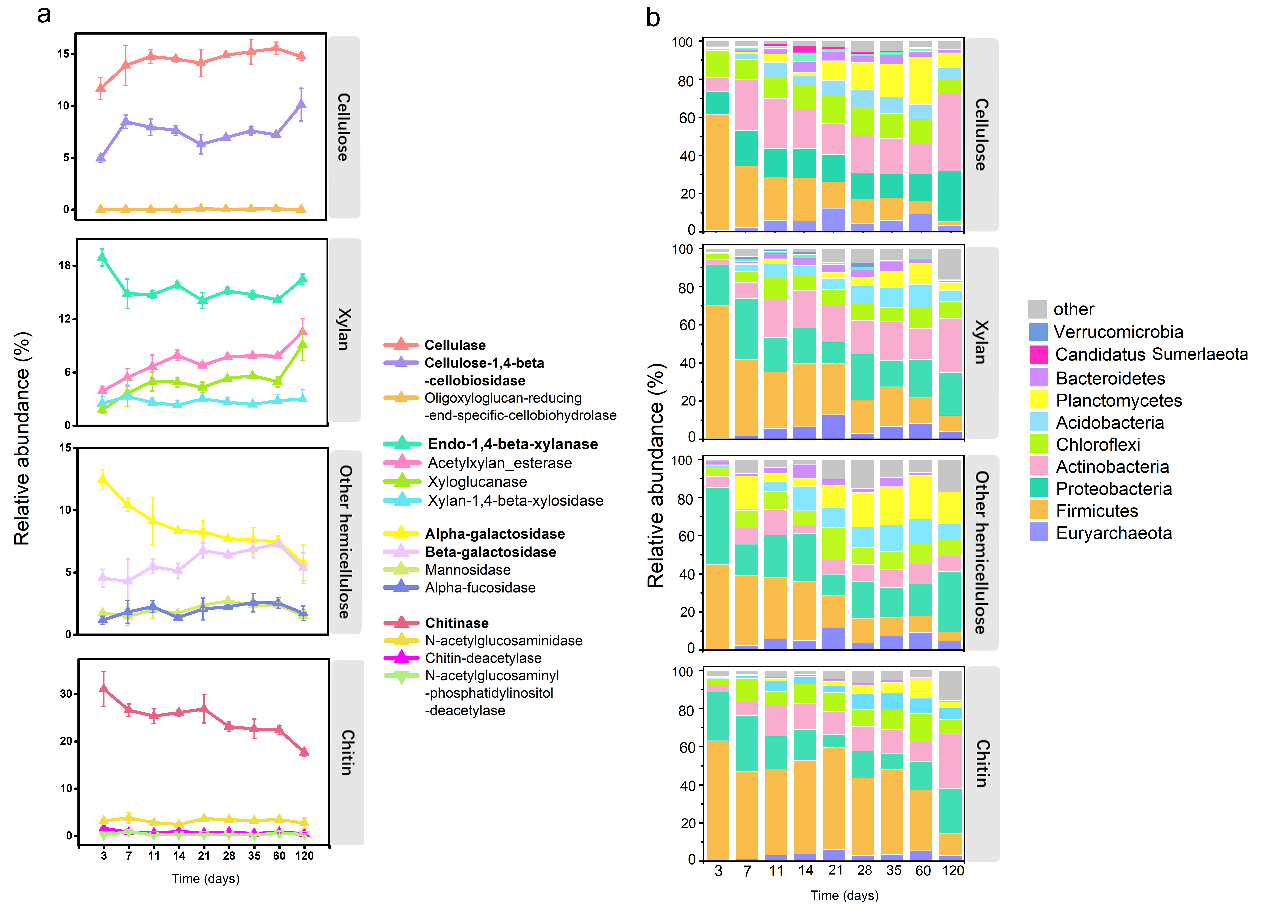
_

**Fig. S5** Metatranscriptomic expression dynamics of genes encoding carbohydrate-active enzymes (CAZymes) that are involved in the breakdown of cellulose, xylan, other hemicelluloses, and chitin (**a**). Changes in the taxonomic composition of the CAZyme transcripts over the 120-day incubation period (**b**). The analysis involved multiple CAZyme families of glycosyl hydrolases (GHs) and carbohydrate-binding modules (CBMs). These CAZyme families are specified in Additional File 2, Table S12.


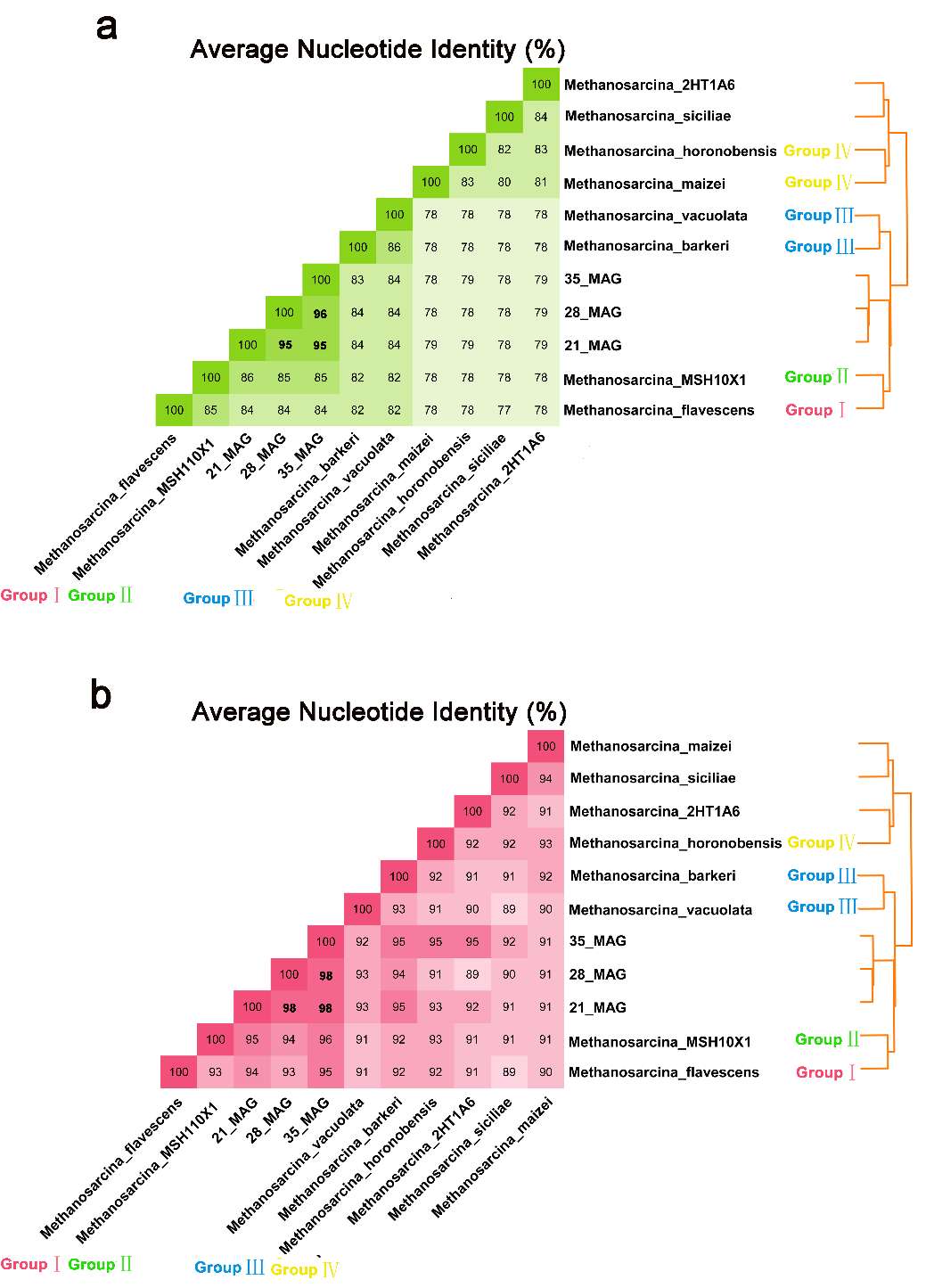


**Fig. S6** Average nucleotide identity (ANI) values calculated for the three *Methanosarcina* MAGs (21, 28, 35) and reference genomes downloaded from the Genome Taxonomic Database (GTDB) **(a)**. Nucleotide sequence identities of the *mcrA* genes present in the same set of MAGs and *Methanosarcina* reference genomes **(b)**.


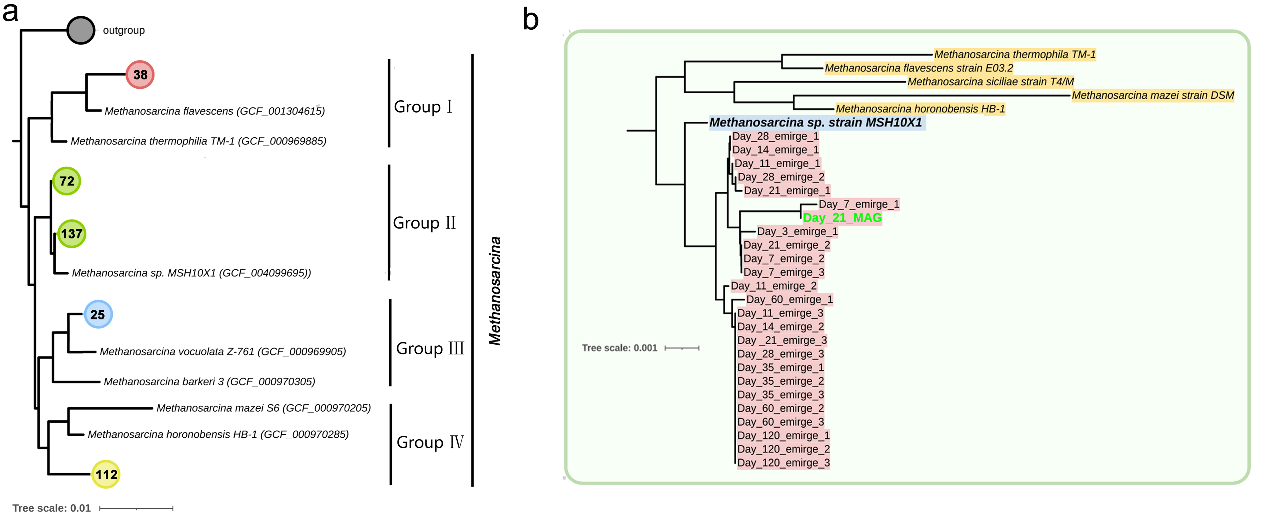


**Fig. S7** Neighbor-joining tree showing the relationship between near full-length 16S rRNA sequences (> 1200 bp) assembled by EMIRGE from the metatranscriptomic datasets (415 sequences) and 16S rRNA gene sequences extracted from the complete *Methanosarcina* reference genomes downloaded from GTDB **(a)**. Neighbor-joining tree showing the position of the near full-length 16S rRNA gene sequence of MAG_21 (1,400 nt) in relation to the 16S rRNA gene sequences extracted from the *Methanosarcina* reference genomes and near full-length 16S rRNA sequences (> 1200 bp) assembled by EMIRGE from the metatranscriptomic datasets.

.


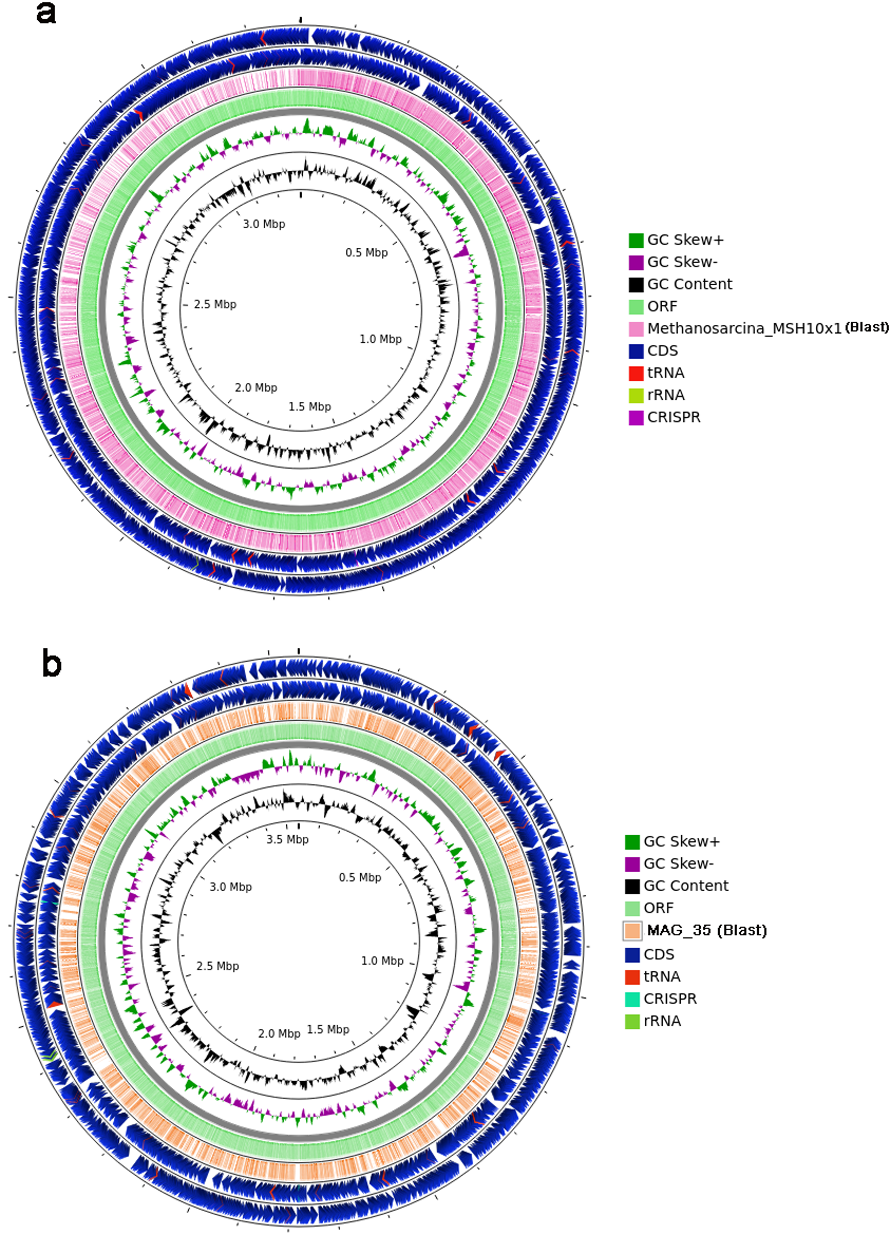


**Fig. S8** Circular genome maps for MAG_35 (**a**) and the most closely related *Methanosarcina* reference genome (strain MSH10X1) (**b**). Circles from the outside to the inside show the positions of protein-coding sequences (blue), tRNA (red) and rRNA genes (green) on the positive (circle 1) and negative (circle 2) strands. Circle 3 shows the positions of BLAST hits detected through BLASTx (with an e-value cut-off of 1e-5) and circle 4 depicts the BLASTx results for the reciprocal search against the genome of strain MSH10X1 (a) and MAG_35 (b). Circles 5 and 6 show GC content and GC skew plotted as the deviation from the genomic average.


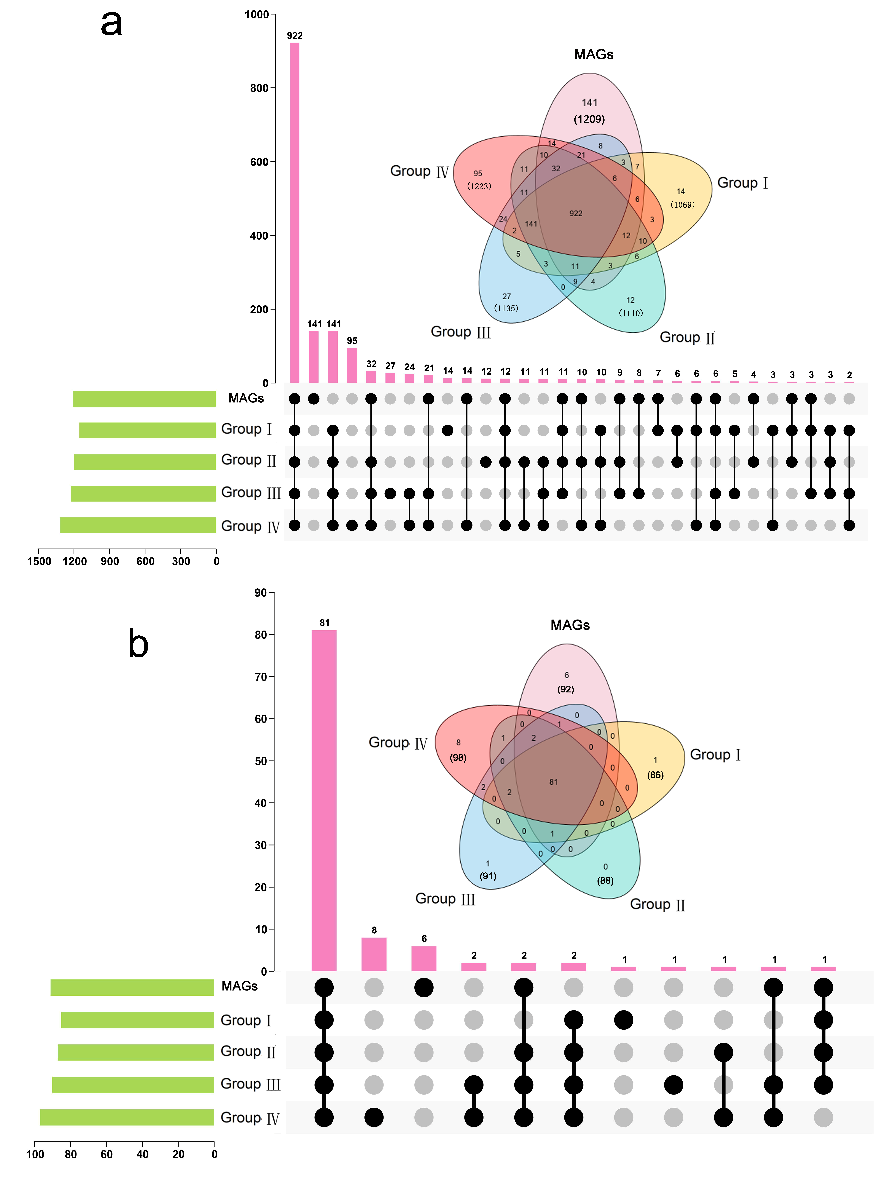


**Fig. S9** Venn diagram showing the distribution of functionally annotated genes among the three *Methanosarcina* MAGs (21, 28, 35) and the reference genomes of *Methanosarcina* Group I (*M. fluorescens*), Group II (strain MSH10X1), Group III (*M. barkeri*), and Group IV (*M. horonobensis*). The bar columns (pink) show the distribution pattern of particular sets of characterized genes among the MAGs and Group I to IV reference genomes. The green columns indicate the total number of predicted genes in the MAGs and Group I to IV reference genomes. The black and grey dots specify presence or absence of this particular set of characterized genes in the *Methanosarcina* MAGs and the Group I to Group IV reference genomes.

Comparative genomics revealed that the three *Methanosarcina* MAGs (21, 28, 35) share their majority of predicted genes with the reference genomes of *Methanosarcina* Groups I to IV (922 characterized genes in common), while they encode 141 unique genes as specified in Table S15 (Additional File 2). The unique genes were associated with the following KEGG level 2 categories: cell wall component biosynthesis, cofactor biosynthesis, membrane transport, energy metabolism, cell motility, and genetic information processing. All the genes encoding essential enzymes for acetoclastic (*K00925*, *K00625*, *K00192*, *K00193*, *K00194*, *K00195* and *K00197*), hydrogenotrophic (*K00200-K00205*, *K11260*, *K11261*, *K00627*, *K01499*, *K00319* and *K00320*), and methanol-dependent methanogenesis *(K14080*, *K04480* and *K14081*) were identified on all three *Methanosarcina* MAGs as specified in Table S16 (Additional File 2).


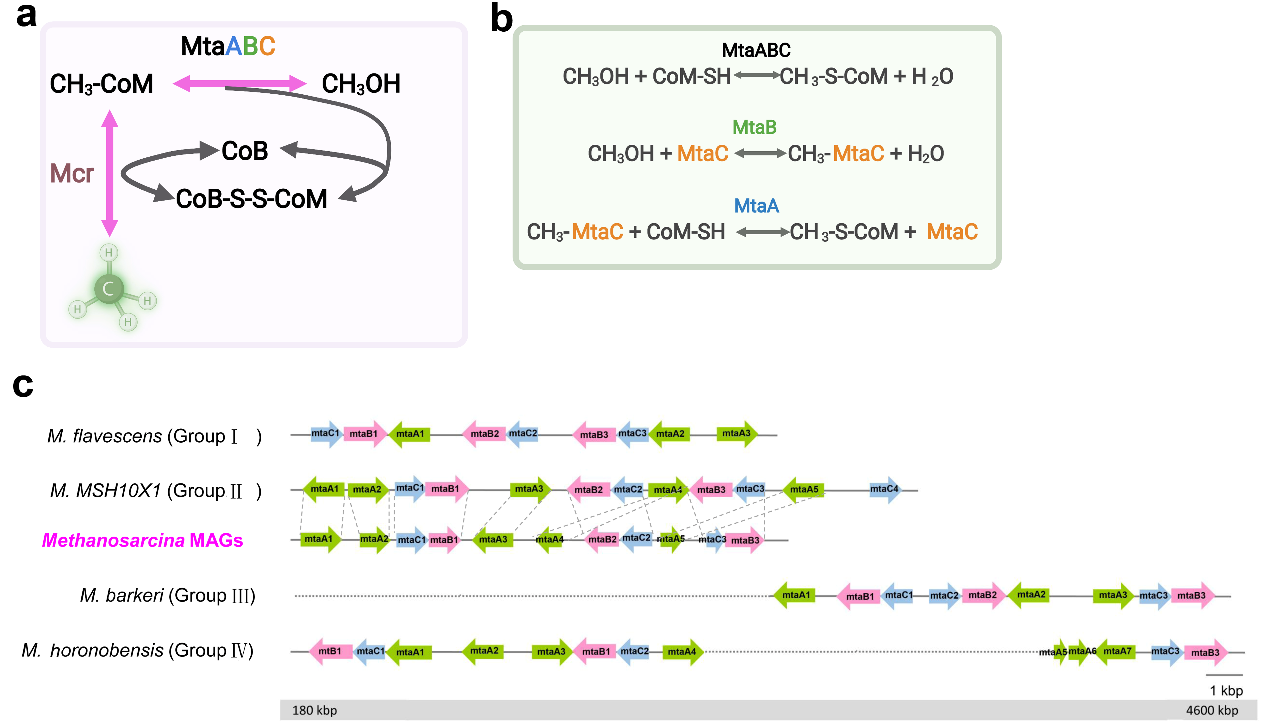


**Fig. S10** Methanol-dependent methanogenesis pathway in the three *Methanosarcina* MAGs (21, 28, 35) and the *Methanosarcina* Groups I to IV reference genomes. *Methanosarcina* populations produce the MtaABC enzyme complex to catalyze the methyl transfer from methanol to CoM to form methyl-CoM (**a** and **b**). Comparison of the genetic organization of the *mta* gene cluster between the three *Methanosarcina* MAGs (21, 28, 35) and the Group I to IV reference genomes **(c)**.

**
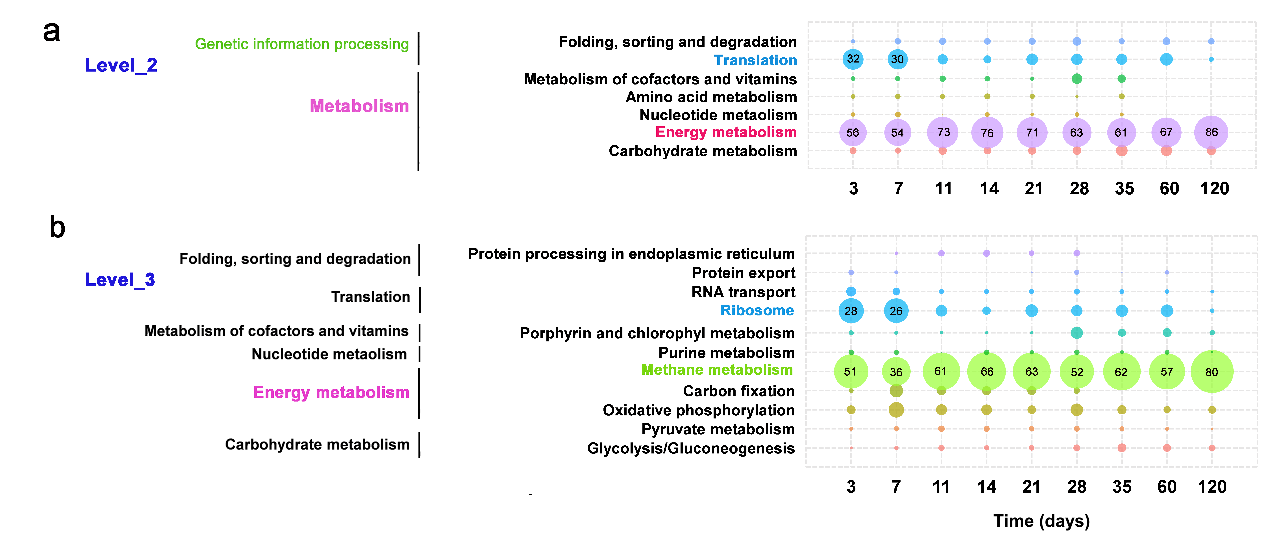
**

**Fig. S11** Relative abundances of mRNA mapped onto the three *Methanosarcina* MAGs (21, 28, 35) and affiliated with particular KEGG categories on level 2 (**a**) and 3 (**b**). The triplicate metatranscriptomes of a given incubation time point were individually mapped onto a particular MAG as follows: (i) triplicate metatranscriptomes generated from RNA of the sampling days 3, 7, 11, 14, and 21 onto MAG_21; (ii) triplicate metatranscriptomes generated from RNA of the sampling day 28 onto MAG_28; and (iii) triplicate metatranscriptomes generated from RNA of the sampling days 35, 60, and 120 onto MAG_35. The relative mapping efficiencies were calculated based on the normalized number of mRNA reads that could be mapped onto each ORF of a given *Methanosarcina* MAG. The dot sizes indicate relative mapping efficiencies.
