# Supplementary material for: Time-shifted expression of acetoclastic and methylotrophic methanogenesis by a single Methanosarcina genomospecies predominates the methanogen dynamics in Philippine rice field soil
Source: Microbiome. 2024 Feb 26;12:39. doi: 10.1186/s40168-023-01739-z (PMC10895765; doi:10.1186/s40168-023-01739-z)
Supplement: Supplementary file 4 — Additional file 3: Supplemental Discussion. [file 40168_2023_1739_MOESM3_ESM.docx]

**Time-shifted expression of acetoclastic and methylotrophic
methanogenesis by a single *Methanosarcina* genomospecies predominates
the methanogen dynamics in Philippine rice field soil**

Xin Li^1#^, Qicheng Bei^1ω^, Mehrdad Rabiei Nematabad^1^, Jingjing Peng^2^,
and Werner Liesack^1*^

*^1^Research group “Methanotrophic Bacteria and Environmental Genomics/
Transcriptomics”, Max Planck Institute for Terrestrial Microbiology,*

*Karl-von-Frisch-Str. 10, Marburg, D-35043, Germany*

*^2^State Key Laboratory of Nutrient Use and Management, College of Resources and Environmental Sciences, National Academy of Agriculture Green Development,*

*Key Laboratory of Plant-Soil Interactions, China Agricultural University,
Beijing, 100193, China.*

**Supplemental Discussion**

**The bacterial community dynamics**

**The bacterial community dynamics**

The complex bacterial community anaerobically degrades organic matter to yield the main methanogenic precursors, including acetate, propionate, butyrate, H_2_, and CO_2_. Like methanogens, the bacterial community composition and activity also showed successional changes during the subsequent stages of organic matter decomposition (Figs. 3, S3).

In particular, the abundance dynamics of the predominant *Geobacteraceae* revealed on the rRNA level and, to a slightly lesser extent, the mRNA level a separation into early (day 3 to 14) and late (days 28 to 60) activity phases, similar as observed for the *Methanosarcinaceae* (Figs. 3, 4). Indeed, the *Geobacteraceae* rRNA abundance was much greater in the Philippine paddy soil than observed in comparable slurry incubations of rice field soils from other geographic locations. In Italian rice field soil, the *Geobacteraceae* abundance ranged, for example, only between 1% and 6% of total community-wide 16S rRNA [1]. Members of the *Geobacteraceae* are able to operate various anaerobic energy-yielding pathways: anaerobic respiration and syntrophic degradation of fermentation intermediates [2]. Upon initiation of our slurry incubations, the nearly immediate activity response of the *Geobacteraceae* (Fig. 3a, b) relates well to the initial reductive phase of rice straw degradation, which is characterized by the limited availability of alternative electron acceptors [3, 4]. Members of the *Geobacteraceae* are metabolically versatile. Their hallmark feature is the ability to reduce insoluble Fe(III) and Mn(IV), but they are also able to utilize a variety of other electron acceptors, such as nitrate, nitrite, nitrous oxide, sulfur, fumarate, and malate [5]. In anoxic rice field soil, alternative electron acceptors are completely exhausted during the first few days after flooding [3]. The detection of *Geobacter*-affiliated mRNA involved in the activity of c-type cytochromes at incubation day 3 corroborates that anaerobic respiration of *Geobacteraceae* members occurred in the very early incubation period (Fig. S4). These cytochromes are involved in the transfer of electrons to Fe(III) or other electron acceptors [6]. Moreover, members of the *Geobacteraceae* are well known for their capacity to transfer electrons extracellularly. Indeed, *Methanosarcina* spp., but also *Methanothrix* spp., are capable of directly accepting electrons from *Geobacteraceae* members for the reduction of carbon dioxide to methane [7, 8]. This direct electron transfer (DIET) may be one likely explanation for the good correspondence between the activity dynamics of *Geobacteraceae* and *Methanosarcinaceae*, as observed on both rRNA and mRNA levels (Figs 3, 4). In addition, DIET by members of the *Geobacteraceae* has been shown to accelerate the syntrophic conversion of butyrate [9]. Given the close correspondence of the transient peak concentration of butyrate with the peak abundances of rRNA and in particular mRNA of the *Geobacteraceae*, it seems reasonable to propose that members of the *Geobacteraceae* were involved in syntrophic butyrate oxidation (Fig. 1a vs. Fig. 3). Another functional interplay between the *Geobacteraceae* and the predominant *Methanosarcinaceae* Group II population may be linked to the anaerobic degradation of lignin and the peak abundance of methylotrophic methanogenesis during the later activity phase. The anaerobic decomposition of lignin and the release of methanol requires the activity of bacterial laccases-like enzymes that anaerobically depolymerize lignin into smaller polymer units to which the enzyme pool of the anaerobic bacteria can get access to decompose them into monomers [10,11]. Among the bacterial community, members of the *Geobacteraceae* are the most promising candidates for being involved in the anaerobic lignin degradation. These bacteria showed not only the expression of putative laccase genes but also their greatest relative expression level during the later phase (days 28 and 35) (Table S21).

Members of the *Peptococcaceae* were previously shown to be involved in syntrophic propionate oxidation [12] and repeatedly detected with high abundance in paddy soils [1,13–15]. A perfect correspondence between the propionate turnover and the mRNA dynamics of both *Peptococcaceae* and *Methanocellaceae* suggests that *Peptococcaceae* members were the key players in propionate oxidation with *Methanocella* spp. being the syntrophic partner (Figs. 1c, 3d, and 4b). In addition, *Peptococcaceae* members may be involved in the degradation of recalcitrant lignin. They not only show a good correspondence of their rRNA and mRNA peak abundances with the expression peaks of methylotrophic methanogenesis but also have been identified in previous research as putative aromatic ring-cleaving bacteria in rice field soil under lignin-degrading methanogenic conditions [16]**.**

Members of the *Anaerolineaceae* and *Acidobacteriaceae* were the two family-level groups that exhibited a steady increase in their relative rRNA and mRNA abundances with incubation time (Figs. 3 c, d). Members of the *Anaerolineaceae* (within the phylum *Chloroflexi*) have the metabolic capacity to degrade alkanes in syntrophic cooperation with *Methanothrix* spp. [17]. This putative functional role in our slurry incubations is supported by the close correspondence between the RNA abundance dynamics of the *Anaerolineaceae* and *Methanotrichaceae*. Both family-level groups exhibit their peak abundances on both rRNA and mRNA levels after the 120-day incubation period (Figs. 3c, d vs. Fig. 4a, b). The significant increase in the metatranscriptomic abundance of *Acidobacteriaceae* on the rRNA level, but in particular on the mRNA level, during the late phase (days 35 to 120) may be due to their oligotrophic life-strategy [18,19] and their ability to hydrolyze recalcitrant plant polysaccharides [20–22].

**References**

1. Wegner C-E, Liesack W. Microbial community dynamics during the early stages of plant polymer breakdown in paddy soil: Metatranscriptomics of rice straw decomposition. Environ Microbiol. 2016;18:2825–42.

2. Röling WFM. The Family *Geobacteraceae*. In: Rosenberg E, DeLong EF, Lory S, Stackebrandt E, Thompson F, editors. The Prokaryotes: *Deltaproteobacteria* and *Epsilonproteobacteria*. Berlin, Heidelberg: Springer; 2014;157–72.

3. Achtnich C, Bak F, Conrad R. Competition for electron donors among nitrate reducers, ferric iron reducers, sulfate reducers, and methanogens in anoxic paddy soil. Biol Fertil Soils. 1995;19:65–72.

4. Chidthaisong A, Conrad R. Pattern of non‐methanogenic and methanogenic degradation of cellulose in anoxic rice field soil. FEMS Microbiology Ecology. 2000;31:87–94.

5. Straub KL, Buchholz-Cleven BE. *Geobacter bremensis* sp. nov. and *Geobacter pelophilus* sp. nov., two dissimilatory ferric-iron-reducing bacteria. International Journal of Systematic and Evolutionary Microbiology. 2001;51:1805–8.

6. Ueki T. Cytochromes in extracellular electron transfer in *Geobacter*. Applied and Environmental Microbiology. 2021;87:e03109-20.

7. Rotaru A-E, Shrestha PM, Liu F, Shrestha M, Shrestha D, Embree M, et al. A new model for electron flow during anaerobic digestion: direct interspecies electron transfer to *Methanosaeta* for the reduction of carbon dioxide to methane. Energy Environ Sci. 2013;7:408–15.

8. Rotaru A-E, Shrestha PM, Liu F, Markovaite B, Chen S, Nevin KP, et al. Direct interspecies electron transfer between *Geobacter metallireducens* and *Methanosarcina barkeri*. Applied and Environmental Microbiology. 2014;80:4599–605.

9. Li H, Chang J, Liu P, Fu L, Ding D, Lu Y. Direct interspecies electron transfer accelerates syntrophic oxidation of butyrate in paddy soil enrichments. Environmental Microbiology. 2014;17.

10. Khan MU, Ahring BK. Lignin degradation under anaerobic digestion: Influence of lignin modifications -A review. Biomass and Bioenergy. 2019;128:105325.

11. Venkatesagowda B. Enzymatic demethylation of lignin for potential biobased polymer applications. Fungal Biology Reviews. 2019;33:190–224.

12. de Bok FAM, Stams AJM, Dijkema C, Boone DR. Pathway of propionate oxidation by a syntrophic culture of *Smithella propionica* and *Methanospirillum hungatei*. Applied and Environmental Microbiology. 2001;67:1800–4.

13. Lueders T, Pommerenke B, Friedrich M. Stable-isotope probing of microorganisms thriving at thermodynamic limits: syntrophic propionate oxidation in flooded soil. Applied and environmental microbiology. 2004;70:5778–86.

14. Gan Y, Qiu Q, Liu P, Rui J, Lu Y. Syntrophic oxidation of propionate in rice field soil at 15 and 30 °C under methanogenic conditions. Appl Environ Microbiol. 2012;78:4923–32.

15. Peng J, Wegner C-E, Bei Q, Liu P, Liesack W. Metatranscriptomics reveals a differential temperature effect on the structural and functional organization of the anaerobic food web in rice field soil. Microbiome. 2018;6:169.

16. Kato S, Chino K, Kamimura N, Masai E, Yumoto I, Kamagata Y. Methanogenic degradation of lignin-derived monoaromatic compounds by microbial enrichments from rice paddy field soil. Sci Rep. 2015;5:14295.

17. Liang B, Wang L-Y, Mbadinga SM, Liu J-F, Yang S-Z, Gu J-D, et al. *Anaerolineaceae* and *Methanosaeta* turned to be the dominant microorganisms in alkanes-dependent methanogenic culture after long-term of incubation. AMB Express. 2015;5:37.

18. Kielak AM, Barreto CC, Kowalchuk GA, van Veen JA, Kuramae EE. The ecology of *Acidobacteria*: moving beyond genes and genomes. Frontiers in Microbiology. 2016;7.

19. Eichorst SA, Trojan D, Roux S, Herbold C, Rattei T, Woebken D. Genomic insights into the *Acidobacteria* reveal strategies for their success in terrestrial environments. Environmental Microbiology. 2018;20:1041–63.

20. Cantarel BL, Coutinho PM, Rancurel C, Bernard T, Lombard V, Henrissat B. The carbohydrate-active enzymes database (CAZy): an expert resource for glycogenomics. Nucleic Acids Res. 2009;37:D233-238.

21. Ward NL, Challacombe JF, Janssen PH, Henrissat B, Coutinho PM, Wu M, et al. Three genomes from the phylum *Acidobacteria* provide insight into the lifestyles of these microorganisms in soils. Appl Environ Microbiol. 2009;75:2046–56.

22. Rawat SR, Männistö MK, Bromberg Y, Häggblom MM. Comparative genomic and physiological analysis provides insights into the role of *Acidobacteria* in organic carbon utilization in Arctic tundra soils. FEMS Microbiol Ecol. 2012;82:341–55.
